# Selection and Characterization of Anti-Dengue NS1 Single Domain Antibodies

**DOI:** 10.1038/s41598-018-35923-1

**Published:** 2018-12-27

**Authors:** Lisa C. Shriver-Lake, Jinny L. Liu, Dan Zabetakis, Victor A. Sugiharto, Cheng-Rei Lee, Gabriel N. Defang, Shuenn-Jue L. Wu, George P. Anderson, Ellen R. Goldman

**Affiliations:** 1 Center for Biomolecular Science and Engineering, US Naval Research Laboratory, 4555 Overlook Ave SW, Washington, DC 20375 USA; 2 Viral and Rickettsial Diseases Department, Naval Medical Research Center, 503 Robert Grant Avenue, Silver Spring, MD 20910 USA

## Abstract

Reliable detection and diagnosis of dengue virus (DENV) is important for both patient care and epidemiological control. Starting with a llama immunized with a mixture of recombinant nonstructural protein 1 (NS1) antigen from the four DENV serotypes, a phage display immune library of single domain antibodies was constructed and binders selected which exhibited specificity and affinity for DENV NS1. Each of these single domain antibodies was evaluated for its binding affinity to NS1 from the four serotypes, and incorporated into a sandwich format for NS1 detection. An optimal pair was chosen that provided the best combination of sensitivity for all four DENV NS1 antigens spiked into 50% human serum while showing no cross reactivity to NS1 from Zika virus, yellow fever virus, tick-borne encephalitis virus, and minimal binding to NS1 from Japanese encephalitis virus and West Nile virus. These rugged and robust recombinant binding molecules offer attractive alternatives to conventional antibodies for implementation into immunoassays destined for resource limited locals.

## Introduction

Dengue fever is a mosquito-borne viral infection and is commonly found in many tropical and sub-tropical countries. Global incidences have increased dramatically over the last decade and it was recently estimated that annually there are 390 million dengue virus (DENV) infections worldwide^1,2^. The virus exists as four antigenically distinct virus serotypes (DENV-1, -2, -3 and -4) under the genus *Flavivirus* in the family *Flaviviridae*. While most infections in humans are asymptomatic or have mild symptoms (75%)^3^, infection can result in dengue fever or a more severe form known as dengue hemorrhagic fever/dengue shock syndrome. Early diagnosis of DENV infection is important to differentiate between dengue fever and other febrile illnesses with similar clinical symptoms, such as malaria, yellow fever, Japanese encephalitis, and chikungunya^4,5^. Current diagnostic testing is focused on detecting the virus or viral nucleic acid (RT-PCR within the first five days) and DENV-specific antibodies, either DENV-specific IgM (several days after onset of symptoms) or DENV-specific IgG^2,6–9^. However, cross-reactivity to other flaviviruses is a major concern with these antibody-response tests^10^.

The DENV nonstructural protein 1 (NS1) is a ~47 kDa glycoprotein which is produced during viral replication and has been identified as an important antigen in DENV infection^10,11^. The NS1 protein is produced by all flaviviruses and is secreted from infected cells during early phases of infection. It is found within one day after the appearance of symptoms in both primary and secondary dengue infections, while antibodies do not peak until after one week following the first symptoms of a primary infection^12,13^. The presence of NS1 antigen in high concentration in patient sera makes it an attractive biomarker of DENV infection. Many types of immunoassays utilizing monoclonal or polyclonal antibodies have been developed for the detection of DENV NS1^14–16^ and a number of these immunoassays are available commercially in the form of ELISA kits or lateral flow rapid assays^5,6,17^.

Our interest in developing single domain antibodies (sdAb) for the detection of DENV NS1 is multifaceted. A survey on DENV diagnostic requirements from US military end users revealed that first responders (self-aid/buddy aid) ideally would like DENV diagnostics with no cold chain requirement; *i.e*. one that could withstand “extreme environmental storage” was identified as an important characteristic of an ideal diagnostic product to first responders^18^. Recognized for providing small and stable binding domains, sdAb are derived from the variable domain of the heavy-chain-only antibodies found in camelids: including camels, llamas, and alpacas^19,20^. They combine the sensitivity and specificity of conventional antibodies with advantages that come from being comprised of only a single domain, such as high physical-chemical stability including heat-resistance, the ability to refold after denaturation, excellent solubility in water, and the capacity to be produced using recombinant technology in good yield^21–24^. Produced using recombinant technology, most often in *Escherichia coli*, sdAb are amenable to the formation of fusion constructs to tailor their integration into a variety of assay formats and sensor systems^25–30^. They can also be modified to improve their biophysical properties; mutagenesis has led to variants with improved protein production and stability, as assessed by the protein’s melting point^31–35^. In addition, one can take what is already a rugged and reliable immunoreagent and create even more robust versions for detection applications in resource-limited areas that lack refrigeration.

A growing number of virus-targeting sdAb have been selected and found to be of promise for both diagnostic and therapeutic applications^36–39^. Fatima *et al*.^40^ selected a llama sdAb against DENV-2 NS1 from a naïve library for incorporation into test strips. Their results using the sdAb compared favorably to the results from monoclonal antibodies in the same type of test kit. This demonstrated the feasibility replacing traditional antibodies with the recombinant sdAb for DENV infection diagnostics; however, cross-reactivity to the NS1 of other variants of DENV and other flaviviruses were not shown.

Herein, we (Naval Research Laboratory) selected and characterized a number of sdAb from an immune library that recognize DENV-1-4 NS1. These binders were found to have a high affinity and specificity and could be utilized to detect clinically relevant levels of DENV-1-4 NS1 when spiked into 50% serum samples. Cross-reactivity towards the NS1 of other flaviviruses was also examined, and a pair of sdAb with good specificity for DENV NS1 was identified. An optimized MagPlex assay incorporating this pair of sdAb was demonstrated to detect DENV NS1 spiked into human serum samples. The reproducibility of this assay was confirmed by the Naval Medical Research Center (NMRC).

## Methods

### Reagents

The NS1 recombinant antigen from the four DENV serotypes as well as from the other flaviviruses was purchased from the Native Antigen Company (Kidlington, UK). Normal human serum was purchased from Valley Biomedical (Winchester, VA). Restriction enzymes and ligase were purchased from New England Biolabs (Ipswich, MA). Unless otherwise specified, chemical reagents were acquired from either Sigma Aldrich (St. Louis, MO), Fisher Scientific (Hampton, NH), or VWR International (Radnor, PA).

### Construction of sdAb Library

A llama (Triple J Farms, Bellingham, WA) was subjected to four rounds of immunization with a mixture of NS1 from all four DENV serotypes (50 µg each NS1). The Triple J Farms Institutional Animal Care and Use Committee (IACUC) reviewed and approved the immunization protocol. All methods were performed in accordance with the relevant guidelines and regulations.

An immune sdAb phage-display library was constructed as described previously^41–43^. Briefly, two weeks following the final immunization, approximately 200 mL blood was drawn from the animal. RNA was isolated from peripheral blood lymphocytes using the QIAamp RNA Blood Mini Kit (Qiagen, Hilden, Germany) according to the manufacturer’s protocol. Purified RNA was converted to cDNA using RT-PCR. The sdAb library was constructed using primers specific for regions flanking the variable heavy domain^20^. The variable domain genes were digested with *SfiI*, purified by Qiaquick gel extraction kit, and cloned into the phage display vector, pECAN21^44^ via a T4DNA ligase ligation reaction with *SfiI*-digested vector and insert at a 3:1 ratio. Ligation products were transformed into electrocompetent XL1-Blue cells (Agilent, Santa Clara, CA) and library diversity was estimated based on a direct count of colony forming units on output plates following ligation/transformation. The sequences of a small sampling were examined to directly assess sequence variability and ensure an open reading frame (ORF) in the majority of clones.

In addition, the whole library was subjected to high throughput sequencing which confirmed sequence diversity and library quality^45^. Sequencing was done on the Illumina MiSeq instrument using “Reagent Kit v3 (600 cycles)”. Paired-end reads were combined with FLASH 1.2.11^46^ and resulted in 11,234,133 sequences. These were translated using MATLAB’s Bioinformatics toolbox, and checked to remove sequences with incorrect ends or having stop/ambiguous codons. This resulted in 6,231,291 ‘good’ sequences (55%). These were processed using the program CD-HIT and using the resources of the DoD High Performance Computing Modernization Program. Clustering^47^ based on 100% identity revealed that the library contains 3,988,881 different sequences, with the average cluster containing 1.56 sequences. Clustering based on 98.46% identity grouped sequences that vary by only 1 or 2 amino acid differences. This revealed 2,527,820 different clusters. Average size was 2.47 sequences.

### Panning

The library was panned against NS1 from DENV-1, DENV-2, and DENV-3. For each variant of DENV NSI, the panning was done using procedures previously described^42–44^. Basically, there were three rounds of panning. The recombinant DENV NS1 variant was coated onto a 96-well plate at 5 µg/mL; next wells were blocked with MPBS (PBS with 4% w/v non-fat powdered milk). Phage displaying sdAb derived from the llama (0.5 mg/mL in 100 µL MPBS) was added to the blocked wells for 1–1.5 hrs. The bound phage was eluted from the plate and used to infect exponential XLI Blue cells for 30 min at 37 °C without shaking. These infected cells were used to amplify the polyclonal phage and rescued by M13K07 helper phage to generate amplified phage. The amplified phage was used in the next round of panning.

A subtractive panning was performed for DENV-2. This protocol is the same as above except the buffer for the phage incubation included the other DENV NS1 variants in solution at 5 µg/mL.

### Monoclonal Phage MagPlex Assay

Each variant of DENV NS1 was immobilized onto a separate set of Luminex MagPlex microspheres using the standard two-step 1-ethyl-3-(3-dimethylaminopropyl)carbodiimide hydrochloride (EDC)/N-hydroxysulfosuccinimide (sulfo-NHS) immobilization protocol recommended by the manufacture. To identify clones that produced phage that bind one or more of the DENV NS1 serotypes, the ability of the phage to bind to MagPlex microspheres coated with each of the DENV NS1 serotypes was evaluated. A microtiter plate of individual clones obtained from either the second or third round of panning were grown up and clonal phage produced. The four DENV NS1 MagPlex microspheres were mixed together and incubated with 10 µL of phage from each well of the phage plate in a corresponding well of a round bottom microtiter plate diluted to a final volume of 100 µL with PBSTB (PBS + 0.05% Tween and 1 mg/mL bovine serum albumin). After one hour, the plate (microspheres) was washed and then the biotinylated goat anti-M13 phage (1 µg/mL) was added to each well for 30 minutes, the plate washed again and incubated with 5 µg/mL streptavidin conjugated phycoerythrin (SAPE). The plate was evaluated on the MAGPIX instrument (Luminex Corp., Austin, TX) to identify the anti-DENV NS1-specific clones.

### SdAb Protein Production

The production and purification of sdAb followed the periplasmic methods previously described^42,48^. Representative sdAb from different sequence families were subcloned into the pET22b expression vector and transformed into Turner (DE3) *E. coli* strain (EMD Millipore, Billerica, MA). To produce an sdAb expressing a GS3K tail, the sdAb sequence was subcloned into the GS3K-pET22b expression vector and transformed into the same expression strain^42,49^. A single colony was grown in 50 mL terrific broth (TB)/Ampicillin (Amp; 100 µg/mL) shake flasks overnight at 25 °C with shaking. The cell mixture was transferred to 450 mL TB/Amp in a shake flask and grown for two hours at 25 °C with shaking. After periplasmic expression induction with 0.5 mM Isopropyl-β-D-1 thiogalactoside (IPTG), the cells continued to grow for an additional two hours. The solution was spun to generate a cell pellet. The cells were suspended and osmotically shocked in 14 mL cold 750 mM sucrose-100 mM Tris pH 7.5 (Tris-sucrose). After suspension, 1 mL 1 mg/mL hen egg lysozyme in Tris-sucrose was added. While shaking, 28 mL of cold 1 mM ethylenediaminetetraacetic acid (EDTA, pH 8) was added dropwise. After the EDTA addition, 0.25 mL cold 5% deoxycholate in water was added. After 30 min of gentle shaking on ice, 1 mL of 500 mM MgCl_2_ was added and the solution continued shaking for 15 minutes. The cell suspensions were then pelleted and the supernatant placed into a 50 mL tube containing 5 mL 10x IMAC (0.2 M Na_2_HPO_4_, 4 M NaCl, 0.25 M imidazole pH 7.5 plus 0.02% sodium azide) with 0.5 mL of Ni-Sepharose high performance resin (GE healthcare). The mixture tumbled for two hours at 4 °C and then was washed with 1x IMAC (0.02 M Na_2_HPO_4_, 0.4 M NaCl, 0.025 M imidazole pH 7.5 plus 0.002% sodium azide). The next day the sdAb was eluted from the resin with 1x IMAC containing 250 mM imidazole and further purified into PBS using an ENrich SEC. 70 10 × 300 mm column and a Bio-Rad Duo-flow System. Concentration and yields were determined from the absorbance at 280 nM. Samples were stored either at 4 °C or frozen.

### MagPlex SdAb Assays

Initially direct binding assays were performed to assess the ability of each sdAb to bind to each of the immobilized NS1 antigens. For this purpose each sdAb was biotinylated using a 10-fold excess of EZ-Link NHS-LC-LC-Biotin for 30 minutes and then the excess biotin was removed using Zeba spin columns with the sdAb concentration determined by absorbance at 280 nM. These biotinylated sdAb (Bt-sdAb) were then added at a range of concentrations to the same sets of MagPlex microspheres with each variant of DENV NS1 immobilized as used for the monoclonal phage MagPlex assays. Following a 30 minute incubation the microspheres were washed and incubated with 5 µg/mL SAPE for 30 minutes, washed, and binding evaluated on the MAGPIX instrument.

To evaluate MagPlex sandwich immunoassays, each sdAb was immobilized onto a separate set of microspheres using the standard protocol, with 30 µL of each set coated and diluted to a final volume of 300 µL following immobilization. These bead sets were then mixed together, using 0.5 µL of each MagPlex bead set for each sample to be tested (≥50 MagPlex beads/set). Using a magnet the microspheres were washed twice with PBST (PBS + 0.05% Tween) and resuspended into the desired volume, 5–10 µL/sample. To generate standard curves, the NS1 being tested was spiked in the first row of a 96-well polystyrene round bottom microtiter plate containing either PBSTB or a 50:50 mix of serum and PBST, then a serial dilution was made down the rows of the plate. The mixture of MagPlex microspheres was added to each well and the plate placed in the dark at 4 °C for 30 min. Next, the plate was washed three times with PBST using a 96 f magnet plate (BioTek, Winooski, VT). The beads were then incubated with 50 µL/well of Bt-sdAb (1 µg/mL diluted in PBSTB). After 30 min, the beads were washed three times and incubated for 15 min with 5 µg/mL SAPE diluted into PBSTB (50 µL/well). After a final two washes, 85 µL of PBST was added to each well and the binding measured on the MAGPIX instrument. The median value obtained by the evaluation of ≥50 microspheres for each set was plotted, and error bars plotted as the standard error of the mean (SEM), which was typically less than ±10% the mean of three replicates. When performing amplified assays, after the first SAPE incubation, the beads were washed three times, and 50 µL/well of Bt-Goat anti-streptavidin diluted to 1 µg/mL in PBSTB, was added and incubated for 15 min. The beads were washed as before, and again 50 µL/well of 5 µg/mL SAPE was added and incubated for 15 min. The beads were then washed a final time and measured as above.

To ensure that the assay was reproducible in the hands of another laboratory, a MagPlex microsphere mix (containing a control set and DD7-GS3K set) and Bt-DD5-GS3K, along with recombinant NS1 from DENV-2 and an aliquot of the normal human serum were sent to NMRC for evaluation under a Simple Letter Agreement. The protocol for generation of the standard curve followed by the NMRC group is similar and included in the Supplementary information.

To determine DENV NS1 positive samples from negative samples, the ratio of the signal to background was determined by dividing the signal from the sample by the signal obtained from the blank (zero) sample. To make a reliable determination of the presence of DENV NS1, a ratio of at least 2 was selected, as this ratio represents a value that provides non-overlapping error bars when both the blank (zero) and samples with detectable amounts of DENV NS1 are plotted with error bars that represent three times the SEM.

### Surface Plasmon Resonance (SPR)

Affinity and kinetics measurements were performed using the ProteOn XPR36 (Bio-Rad). Lanes of a general layer compact (GLC) chip were individually coated with DENV NS1 of each serotype or NS1 of West Nile virus (WNV) or Zika virus (ZIKV). Immobilization of the proteins were performed using dilution to 20 µg/mL in 10 mM acetate buffer pH 5.0 and attached to the chip following the standard 1-ethyl-3-(3-dimethylaminopropyl)carbodiimide hydrochloride (EDC)/ N-hydroxysulfosuccinimide (sulfo-NHS) coupling chemistry available from the manufacturer. Binding kinetics of each sdAb was tested at 25 °C by flowing six concentrations typically varying from 300 to 0 nM at 100 μL/min for 90 s over the antigen coated chip and then monitoring dissociation for 600 s. Following each run, the chip was regenerated by flowing 0.085% phosphoric acid (~pH 3.0) across the surface for 18 s. Data analysis was performed with ProteOn Manager 2.1 software, corrected by subtraction of the zero antibody concentration column as well as interspot correction. The standard error on the fits was less than 10%. Binding constants were determined using the Langmuir model built into the analysis software. Similar assays were performed where selected sdAb were immobilized in a like manner to the GLC sensor chip and then tested for their ability to bind dilutions of the various NS1 proteins.

### Measurement of Melting Temperature (Tm) by Fluorescent Dye Melt Assay and Circular Dichroism (CD)

The Fluorescent dye-based melting assay was performed as described previously^43^. Each sdAb was first diluted to a concentration of 500 µg/mL in a final volume of 20 µL PBS. Next Sypro Orange dye was added with a dilution of 1:1000. Samples were measured in triplicate using a StepOne Real-Time PCR machine (Applied Biosystems, Foster City, CA). The heating program was run in continuous mode from 25–99 °C at a heating rate of 1% (~2 °C per minute), and data was recorded using the ROX filter. The melting point was determined to be the peak of the first derivative of the fluorescence intensity.

CD was performed using a Jasco J-815 Spectropolarimeter, following a protocol the same as used in prior work^42^. The sdAb samples were diluted to 22 µg/mL in deionized water and placed in a quartz cuvette with 1 cm path length and CD was measured at an ultraviolet wavelength between 200 and 210 nm. The sdAb samples were heated from 25 °C to 95 °C at a rate of 2.5 °C/min and then cooled back to 25 °C at the same rate.

## Results and Discussion

A phage display library of sdAb was derived from a llama immunized with a cocktail containing recombinant versions of the NS1 antigen from the four DENV serotypes. Quality of the library was confirmed through high throughput sequencing as described in the methods.

The sdAb specific for DENV NS1 were selected by panning the phage display library on NS1 immobilized onto microtiter plates. Standard pannings were performed with three of the four variants (NS1 from DENV-1, -2, and -3) individually adsorbed onto wells of a 96-well plate. In addition, a subtractive panning protocol was performed for DENV-2 NS1, where phage was incubated with NS1 of DENV-1, -3, and -4 present in solution. For each of the four panning experiments, 32–48 individual clones eluted from rounds 2 and/or 3 were grown in a microtiter plate and phage produced. These phages were then evaluated in a direct binding assay using a MagPlex assay to identify NS1 binders^50^. The DENV-1-4 NS1 antigens were each immobilized to separate sets of magnetic microspheres. These sets along with control sets were incubated with aliquots of phage from each prospective clone. Phages that demonstrated specific binding to any of the NS1-coated microspheres were sequenced. These clones fell into five sequence families, two of which consisted of only one member each. Protein sequences are shown in Supplementary Fig. S1.

**Figure 1:**
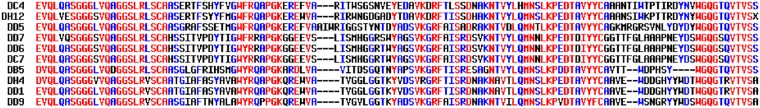
Protein sequences of DENV NS1 binding sdAb that were characterized further. Clones were chosen that span the sequence families isolated in the panning experiments. DC7 and DD6 were isolated from different panning experiments but have identical amino acid sequences. Sequences were aligned using Multalin^57^.

A total of nine unique clones and one duplicate clone from the five sequence families isolated through the panning procedures were cloned into the pET22b expression vector for production and characterization of the soluble sdAb. Protein sequences of the clones that were further characterized are shown in Fig. 1.

After protein production, the sdAb were evaluated for their ability to bind the DENV-1-4 NS1 via the same MagPlex direct binding assay that was used to identify binding clones expressed on phage. However, this time the sdAb were biotinylated, obviating the need for a biotinylated secondary antibody (Bt-M13-IgG) that was used for the phage binding assay. The results of this experiment (Supplementary Fig. S2) indicated that many sdAb showed substantial selectivity towards the various DENV-1-4 serotypes. For instance, DD1 bound best to DENV-2 and DENV-4 NS1, while DB5 showed only binding to DENV-3 NS1. Just as important, most of the clones showed very little nonspecific binding to unrelated targets.

Most of the sdAb showed binding to NS1 from all four DENV serotypes, and sdAb belonging to the three multi-membered sequence families were identified through selection on NS1 from two or three of the DENV serotypes. However, panning on NS1 from DENV-3 yielded DB5, which is specific for this serotype. Using a subtractive panning on DENV-2, which used NS1 of DENV-1, -3, and -4 in a phage blocking solution in an attempt to obtain isotype specific binders, did not yield binding phage specific for the NS1 from DENV-2. The four DENV NS1 antigens are highly homologous (Supplementary Fig. S3) so it is not surprising that the majority of isolated sdAb were not serotype-specific. Potentially further screening of phage eluted in the second and third rounds could reveal other specific binding clones within our selected phage. The fact that all but one of the selected sdAb was not serotype-specific was perhaps a function of our immunization strategy, as our library was derived from one animal which had been immunized with a mixture of NS1 antigen from all four serotypes. If obtaining serotype-specific sdAb were desired, a better strategy might be to immunize four different llamas, each with NS1 from a single DENV serotype. Other researchers have isolated conventional antibody binding domains that specifically recognize NS1 from each of the four serotypes using methods such as combining the use of a non-immune library with a subtractive panning method^51^.

The initial MagPlex binding study was followed up by measuring the binding affinity for each clone to each of the DENV-1-4 NS1 antigens by SPR (Table 1; Supplementary Fig. S4). The results obtained from the MagPlex direct binding assay were mostly in agreement with the binding constants determined by SPR, such as the higher affinity obtained for DD1 binding to NS1 from DENV-2 and -4, and high specificity of DB5 for DENV-3. However, in other cases much better binding was observed for the SPR results than those obtained via the MagPlex assay. For instance, DD5 appeared to be a much better binder as measured by SPR than when looking at the MagPlex direct binding data. Clearly, both methods can be skewed by the need to immobilize the NS1 on the surface, and for the MagPlex assay the sdAb was also chemically modified by the addition of multiple biotins. Attachment to surfaces, and in the case of SPR, the need to regenerate the surface, can obscure or denature the epitope on the NS1, and likewise for the MagPlex assay, the addition of a biotin near the CDR regions of the sdAb can limit or alter its binding ability.

**Table 1:** Melting Point (Tm) and dissociation constants (KD) of the sdAb.

SdAb	Tm °C	KD (nM)			
		DENV-1	DENV-2	DENV-3	DENV-4
DH12	68	14.4	9.8	12.4	20.9
DD5	65	2.8	4.5	3.2	24.0
DC4	64	62.0	42.1	31.6	43.0
DD7	59	5.4	20.3	7.0	2.4
DD6	53	7.6	2.0	1.1	2.5
DC7	52	9.1	2.6	1.2	2.2
DB5	65	NBO	NBO	4.4	NBO
DH4	55	10.3	8.5	23.8	8.5
DD1	61	28.5	15.0	85.8	7.8
DD9	59	18.0	6.7	21.8	34.8

The Tm was determined via the fluorescent dye melt assay and the KD for each sdAb determined from the binding association and dissociation to immobilized NS1 of each variant. NBO denotes that no binding was observed.

To eliminate immobilization of antigen effects in the SPR results, six of the clones, representing four sequence families, were examined wherein the sdAb was immobilized to the sensor chip and each of the DENV-1-4 NS1 antigens were flowed over the chip to measure the apparent binding affinity (Table 2; Supplementary Fig. S5). In this case, we refer the measured K_D_ as an apparent affinity, since the recombinant NS1is known to form aggregates which can decrease the observed off rate due to the avidity of the binding interactions. Nonetheless, these results confirmed that most of the sdAb examined bind to NS1 of all four serotypes, albeit with varying affinities. Only DB5 was found to be specific to NS1 from DENV-3 alone, with no binding to the other three.

**Table 2:** Estimated dissociation constants (KD) of the immobilized sdAb.

SdAb	KD (nM)			
	DENV-1	DENV-2	DENV-3	DENV-4
DD5	4.2	0.9	25.1	6.2
DD7	1.7	1.5	2.4	1.1
DC7	0.8	0.6	1.7	0.7
DB5	NBO	NBO	1.6	NBO
DD1	2.2	2.3	17.7	0.6
DD9	2.7	3.5	3.1	7.1

The KD was determined for six of the sdAb immobilized onto an SPR chip for the binding to recombinant NS1 from each variant. These value are estimates based on the concentration of NS1, which since it is not monomeric are at best relative numbers for purpose of comparison. NBO denotes that no binding was observed.

In addition to affinity measurements, one of the parameters obtained for each clone was its melting temperature (Tm) as determined by a fluorescent dye melt assay (Table 1). The Tm for these sdAb range from a low of 52 °C for DC7 to a high of 68 °C for DH12. These Tms are in the typical range for sdAb previously obtained, but not particularly remarkable, as we have obtained clones with a natural Tm of 85 °C^52^, and can engineer Tm increases of up to 20 °C into sdAb by virtue of selected stabilizing point mutations and the addition of a second disulfide bond^41,42^.

Based on the MagPlex direct binding assay, eight sdAb clones, including at least one representative from each sequence family, were selected to be tested as tracers in a MagPlex sandwich immunoassay using all the sdAb clones immobilized on individual sets of magnetic microspheres (Supplementary Fig. S6). The results of this experiment indicate the capture-tracer pair of DD7/Bt-DD1 gave the most sensitive detection of NS1 from each of the four DENV serotypes.

Next, we down-selected to six of the biotinylated tracer sdAb and tested their specificity for binding NS1 from DENV (tested as a mixture of serotypes) (Supplementary Fig. S7) versus the NS1 from related flaviviruses: Zika virus (ZIKV), yellow fever virus (YFV), Japanese encephalitis virus (JEV), West Nile virus (WNV), and tick-borne encephalitis virus (TBEV) (Supplementary Fig. S7). The NS1 variants all show homology to the NS1 from the DENV serotypes; an alignment of the sequences of these NS1 proteins is shown in Supplementary Fig. S8. None of the DENV sdAb bound to NS1 from ZIKV, YFV, JEV, or TBEV. However, cross reactivity towards NS1 from the WNV was observed. The only tracer sdAb that showed good binding to DENV with no appreciable binding to WNV was DD5. Thus, even though sandwich assays using DD1 as a tracer were more sensitive, DD5 was used in further testing in an effort to develop the most DENV specific assay. The binding patterns of DD7 and DD5 strongly suggest that they recognize different epitopes on the NS1 antigen. The binding epitope of these sdAb could be determined in the future using methods such as linear peptide epitope mapping and can also take advantage of the ability to produce and purify fragments of NS1^53,54^.

**Figure 2:**
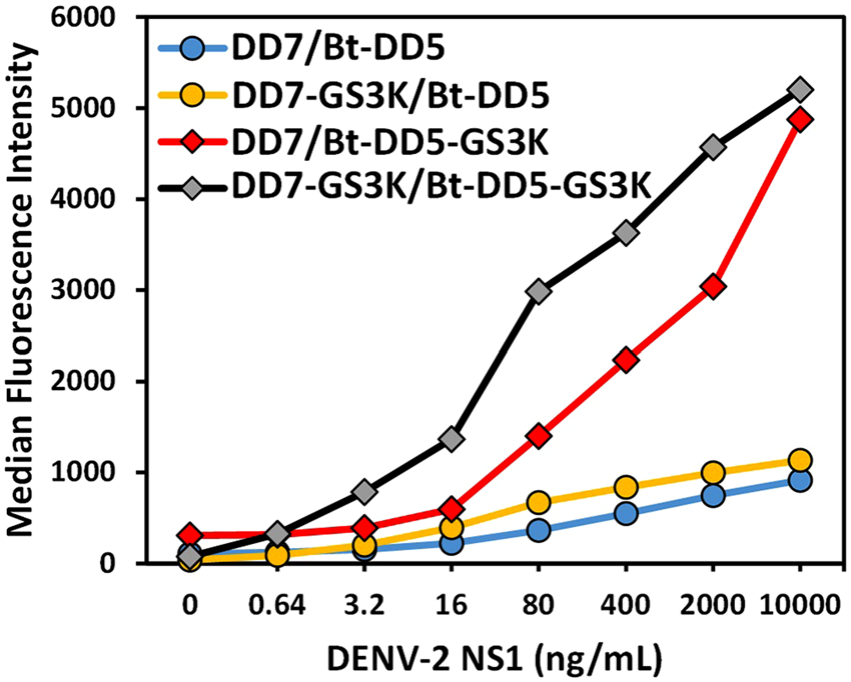
Sandwich assays using sdAb with and without the GS3K tail. Comparison of sdAb with and without GS3K tails as the capture and tracer in MagPlex assays for the detection of NS1 from DENV-2. Each data set is denoted as “capture/tracer”.

Prior to testing DENV NS1-spiked serum samples, we elected to optimize both the capture DD7 and tracer DD5 sdAb by the addition of a C-terminal GS3K tail (amino acid sequence: GGGGSGGGGSKKK), which previously was found to both help orient the sdAb on the surface when immobilized, as well as giving additional preferential sites for biotinylation^42,49^. Figure 2 shows the comparison of sandwich assays for DENV-2 NS1 with the sdAb DD7 and DD5 plus and minus the GS3K tail. The addition of the tail provided a signal improvement for the immobilization of DD7, while the improvement realized in terms of both signal level obtained and limit of detection achieved by the addition the GS3K tail to DD5 for biotinylation was even more dramatic. Tms of the two GS3K variants were measured by CD which was also used to determine the ability of the variants to refold after heat denaturation. The DD7-GS3K clone’s Tm and refolding were measured at 50 °C and 79% refolding while DD5-GS3K’s Tm was 70 °C with 55% refolding.

**Figure 3:**
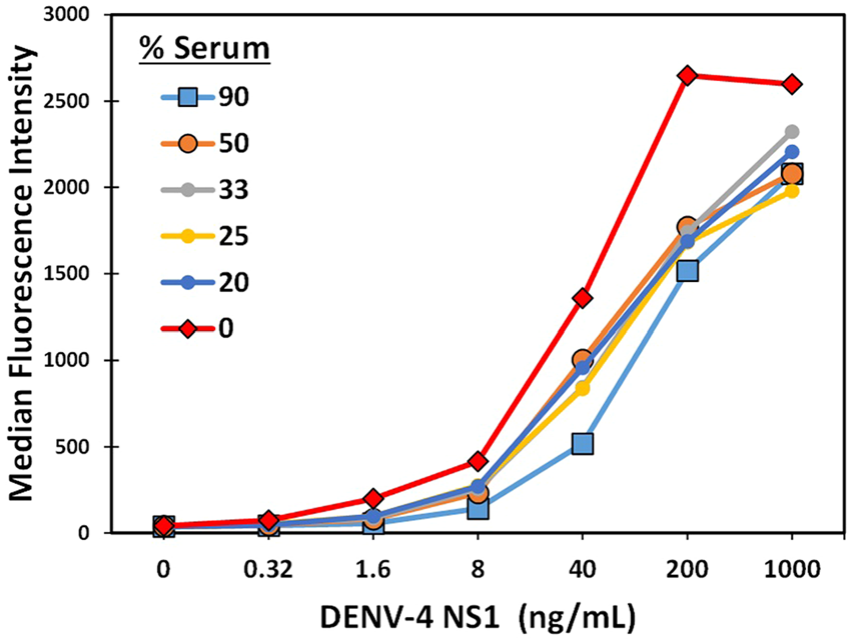
MagPlex sandwich assays to evaluate optimal serum dilution. DENV-4 NS1 was spiked into commercial normal human serum at the percentage indicated. The MagPlex bead set DD7-GS3K and Bt-DD5-GS3K were used in the evaluation.

Using this pair, DD7-GS3K and Bt-DD5-GS3K, the optimal serum dilution to use for the testing of the spiked samples was evaluated (Fig. 3). These data indicated that a 50% serum dilution was sufficient, as further dilutions did not improve the signal levels obtained, thus 50% serum level was selected for all the following spiked serum tests.

**Figure 4:**
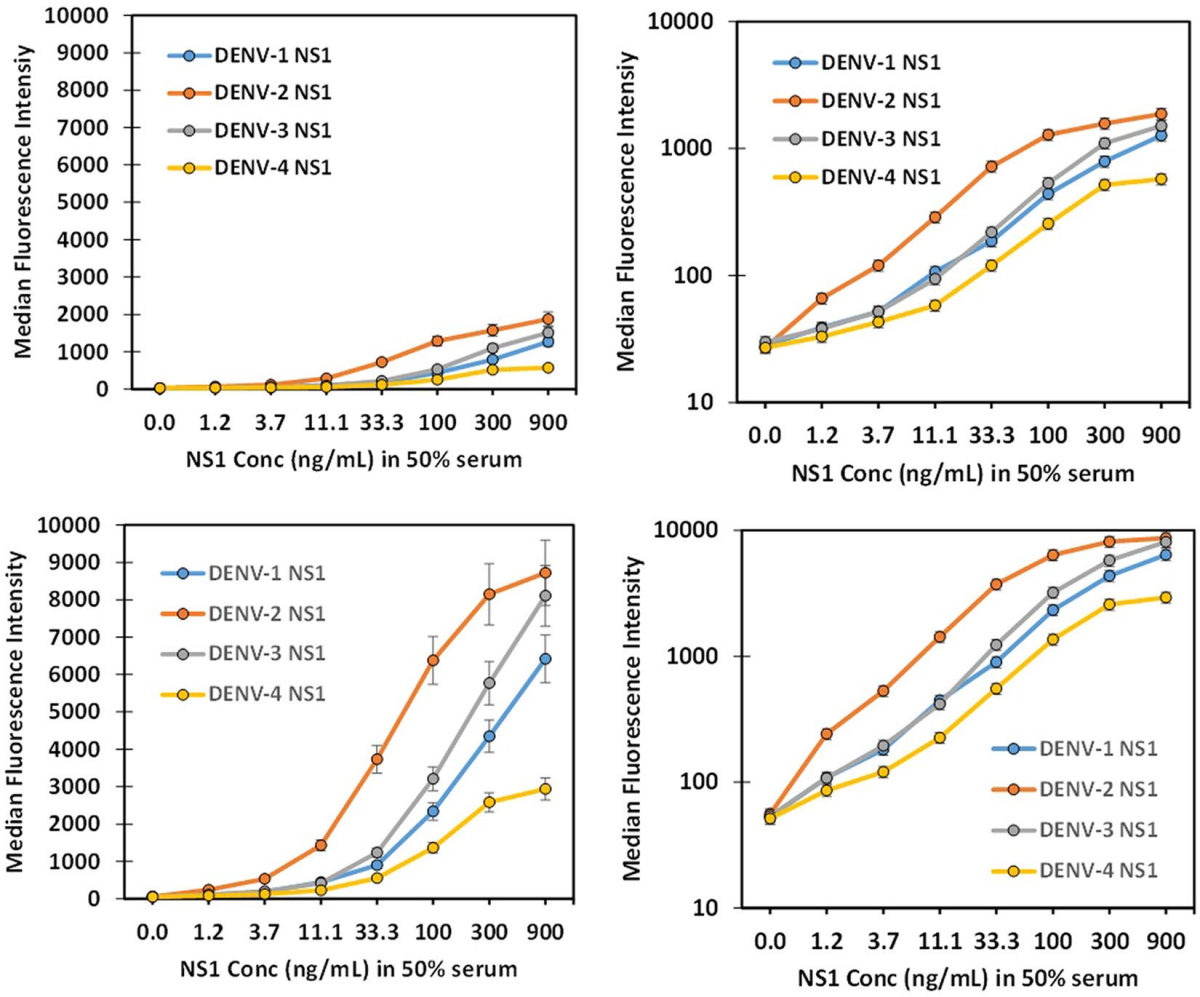
Comparison of standard and amplified DENV NS1 MagPlex assay in 50% serum. Dilutions of each DENV NS1 variant was prepared in 50% serum and then tested either using the standard (top panels, linear plot left and log plot right) or amplified MagPlex assay (bottom panels, linear plot left and log plot right) using DD7-GS3K as the capture sdAb immobilized on the MagPlex beads and Bt-DD5-GS3K as the tracer.

To further enhance our sensitivity in the 50% serum sample, the standard MagPlex assay was compared to an amplified assay^55^, where the first incubation with SAPE, is followed by Bt-goat anti-SA, and then the SAPE incubation is repeated. As Fig. 4 shows, amplification substantially increased signal level and improved the limit of detection. When used in conjunction with conventional antibodies, the MagPlex amplification assay was shown to increase signal levels but did not improve the signal to background ratio. However, for smaller tracers, such as sdAb, that can carry less biotin and provide few binding sites for SAPE on the base layer, amplification does appear beneficial^56^, as it was here.

**Figure 5:**
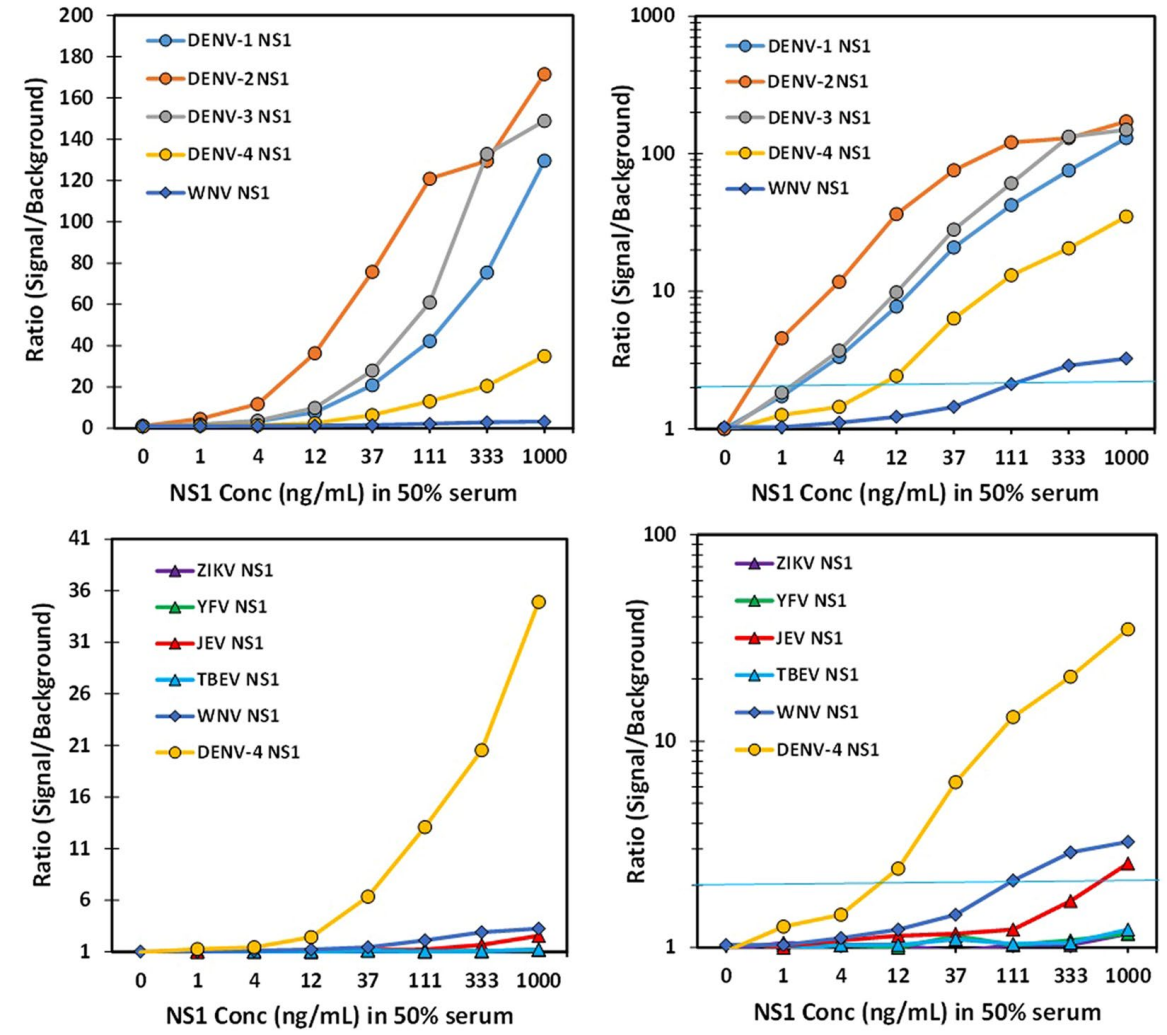
MagPlex detection of DENV NS1 versus NS1 from other flaviviruses. Dilutions of NS1 were prepared in 50% serum and evaluated using the amplified MagPlex assay with DD7-GS3K as the capture and Bt-DD5-GS3K as the tracer sdAb. The top panels (linear plot left and log plot right) show detection of NS1 from all four DENV serotypes versus NS1 from WNV. While the bottom panels (linear plot left and log plot right) show the comparison of signal produced by NS1 from other flaviviruses versus the signal produced by DENV-4 NS1. The blue line in the right panels represents a signal to background ratio of 2; a value greater or equal to 2 was defined as a positive.

Dose response curves for the detection of DENV-1-4 NS1 spiked into 50% serum as well as NS1 for ZIKV, YFV, JEV, WNV, and TBEV were developed using the DD7-GS3K and Bt-DD5-GS3K pair and the MagPlex amplified assay (Fig. 5). For these assays we determined the ratio of the signal measured at each NS1 concentration to the signal obtained by the blank (no NS1) sample; a ratio of 2 was set as our detection limit. The best sensitivity was observed for DENV-2 NS1 (<1 ng/mL), while DENV-1&3 NS1 were both <4 ng/mL, and DENV-4 NS1 was the least sensitive, at ~12 ng/mL. The specificity was very high, showing no binding to ZIKV, YFV, and TEBV, while there was some binding at higher concentrations of WNV and a trace at the highest concentrations of JEV.

To ensure that the assay was reproducible in the hands of another laboratory, a MagPlex microsphere mix (containing a control set and DD7-GS3K set) and Bt-DD5-GS3K, along with recombinant NS1 from DENV-2 and an aliquot of the normal human serum were sent to the NMRC for evaluation under a Simple Letter Agreement. Measurements were done in duplicate and detection was observed down to the lowest dilution, 1.4 ng/mL, with a signal to background ratio of 3 (Supplementary Table S1).

**Figure 6:**
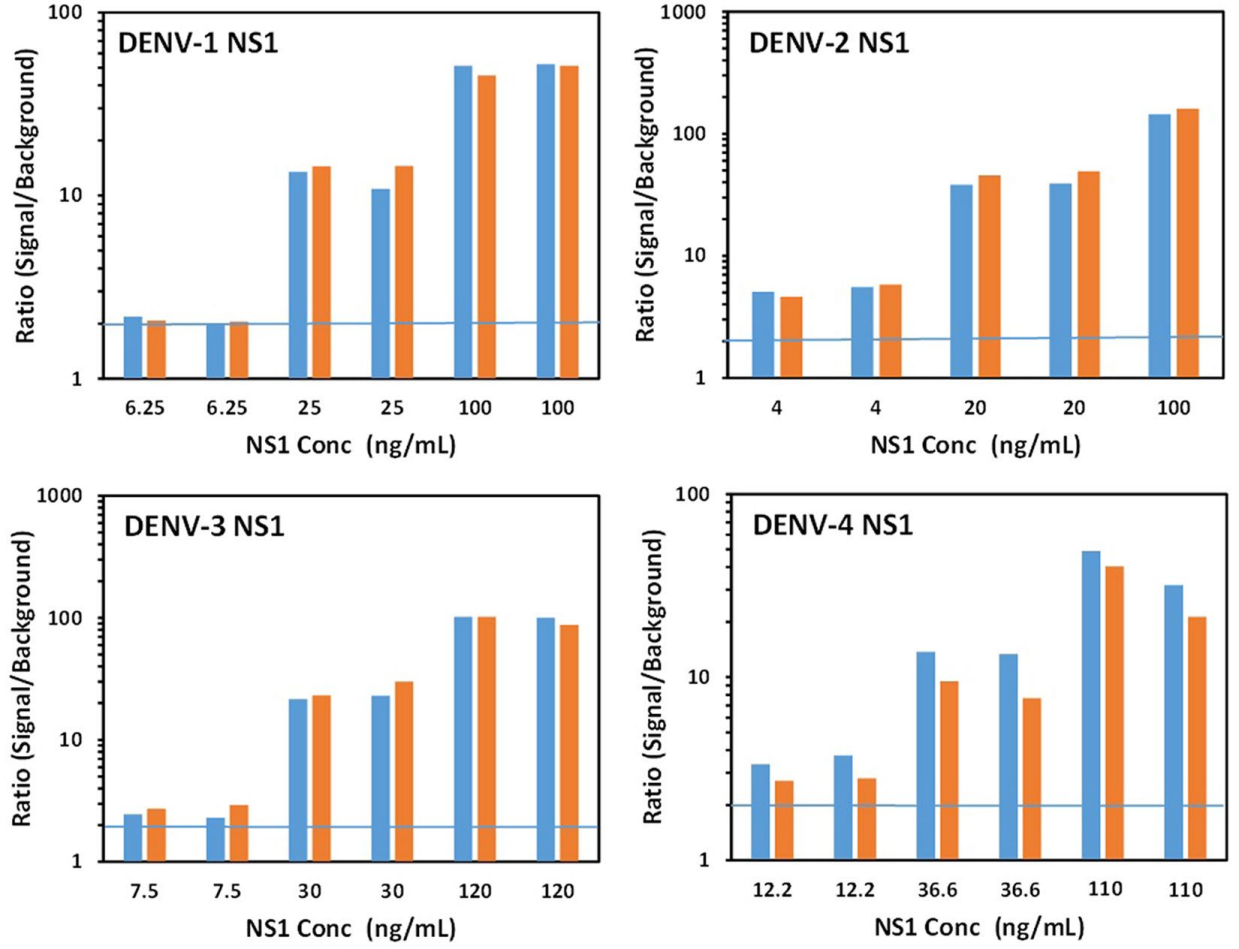
Blinded unknown samples of DENV NS1 spiked into serum samples. The sample concentrations are shown though the assay and analysis were performed blind in random order. Thirty two samples including 9 blanks were prepared and randomly ordered. The samples were then split and evaluated in two separate plates using the MagPlex amplified assay with DD7-GS3K as the capture and Bt-DD5-GS3K as the tracer sdAb. Shown are the results for each NS1 positive sample tested in duplicate sorted by concentration and type. All 9 blanks (not shown) were determined to be negative, with ratios ranging 0.925 to 1.44, while all the unknown samples containing NS1 were identified as positive with ratios of 2.0 or greater. All results in the order tested are shown in Supplementary Fig. 9.

As a further evaluation, the ability of this assay to provide DENV detection in a blinded test format was assessed (Fig. 6 and Supplementary Fig. S9). It is often noted that many assays report remarkable levels of detection when the presence of the target is known, but obviously that is not the case for real patient samples. To test if this assay could correctly identify DENV positive and negative samples, 32 samples (which included nine blanks) were prepared and randomly ordered. The samples were then split and evaluated in two separate plates using the MagPlex amplified assay. All nine blanks were determined to be negative, with ratios ranging 0.925 to 1.44, while all the unknown samples containing NS1 were identified as positive with ratios of 2.0 or greater, with both plates giving similar results.

In conclusion, we have demonstrated the ability of selected sdAb to detect NS1 from the four DENV serotypes spiked into 50% normal human serum using a MagPlex amplified assay. The sdAb pair chosen has good specificity for NS1 from DENV versus NS1 from related virus. Next, it will be necessary to evaluate this assay with well-characterized clinical samples and evaluate the ability of these reagents to withstand harsh storage conditions, prior to transitioning to field testing.

## Electronic supplementary material

Supplementary information file

## Data Availability

Data is presented within the manuscript and the Supplemental Materials.
