# Supplementary material for: Selection and Characterization of Anti-Dengue NS1 Single Domain Antibodies
Source: Sci Rep. 2018 Dec 27;8:18086. doi: 10.1038/s41598-018-35923-1 (PMC6308234; doi:10.1038/s41598-018-35923-1)
Supplement: Supplementary file 1 — Supplementary information file [file 41598_2018_35923_MOESM1_ESM.pdf]

Supplementary Information

Selection and Characterization of Anti-Dengue NS1 Single Domain Antibodies

Lisa C. Shriver-Lake[1], Jinny L. Liu[1], Dan Zabetakis[1], Victor A. Sugiharto[2], Cheng-Rei Lee[2], Gabriel N. Defang[2], Shuenn-Jue L. Wu[2], George P. Anderson[1], and Ellen R. Goldman[1]*

[1]Center for Biomolecular Science and Engineering, US Naval Research Laboratory, 4555 Overlook Ave SW, Washington, DC 20375, USA

[2]Viral and Rickettsial Diseases Department, Naval Medical Research Center, 503 Robert Grant Avenue, Silver Spring, MD 20910, USA

*Corresponding author; Email: ellen.goldman@nrl.navy.mil, phone: 202-404-6052

METHOD USED TO PRODUCE DATA IN SUPPLEMENTAL TABLE S1

MagPlex SANDWICH IMMUNOASSAYS

Reagents/Supplies:

- Mixed MagPlex bead (microsphere) solution (includes experimental and control bead sets)
- PBST- Phosphate buffered saline with 0.05 % Tween (for washes)
- PBSTB- PBST and 1 mg/ml BSA (PBSTB) (for reagent dilutions)
- Serum
- Antigen DENV-2 NS1 –(in Serum at 178 ug/mL)
- Biotin-labelled sdAb (Bt-DD5-gs3k)
- Streptavidin phycoerythrin (SAPE)
- Biotin-labelled goat anti-streptavidin (Bt-goat α SA)
- Microtiter plate – 96 well polyethylene round bottom plate
- Magnetic plate similar to 96f magnet (BioTek, Winooski, VT, USA)
- Magnetic holder for eppendorf tube

Protocol for control assay demonstrating the function of the sdAb for DENV NS1 detection:

For the dose response assay of DENV-2 NS1 in the round bottom polypropylene microtiter plate (low binding).

1. Bead preparation: Make a 1:10 dilution of the mixed bead mixture in PBSTB in a volume equal to at least 10 µl/sample in an Eppendorf tube (i.e. use 1 µl of bead mix stock/sample).
2. Add 20 µl of the DENV-2 NS1 in Serum (178 ng/mL) to Row A of Columns 1 and 2.
3. Add 10 µl of Serum in Rows B-H of Columns 1 and 2.
4. Make serial dilutions of the DENV-2 NS1 by moving 10 µl down from Row A for both columns to B, mix and repeat to row C, etc, down to row G. Leave H as the blank.
5. Add 10 µl of microspheres (make sure to resuspend right before use, as they settle rapidly).
6. Place plate in the dark (or semi-dark) at 4°C for 30 min. Darkness is needed to reduce photobleaching of beads.
7. Wash the plate by placing the plate on a 96f magnet (BioTek, Winooski, VT, USA; or similar) and washing 2 times with PBST. To wash the plate, 150 µl PBST is added to sample in the plate. While holding on magnet, discard liquid. Repeat for 2nd wash. Use magnet only for wash steps.
8. Add 50 µl /well of 1 µg/mL Bt-sdAb (Bt-DD5-gs3k). This is prepared by diluting into required volume of PBSTB to prepare for all wells. Incubate 30 min at 4°C and in dark.
9. Wash the beads 2 times as above.
10. Add 50 µl/well of 5.0 µg/mL streptavidin conjugated phycoerythrin (SAPE), to prepare dilute 1 mg/mL stock 1:200 in PBSTB to form the first layer of the fluorescent sandwich assay. The beads are incubated for 15 min at 4°C and in dark.
11. Wash the beads 2 times as above.
12. Add 50 µl/well 1 ug/ml Bt-Goat anti-streptavidin (in PBSTB) and incubate for 15 min at 4°C and in dark.
13. Wash the beads 2 times as above.
14. Add again 50 ul/ml 5.0 µg/mL SAPE (in PBSTB) to wells and incubate for 15 min at 4°C and in dark.
15. Wash the beads 2 times as above.
16. Add 85 µl of PBST is added to each well
17. Measure fluorescence (binding) on the MAGPIX instrument (Luminex Corp., Austin, TX, USA). The median value obtained by the evaluation of ≥ 50 microspheres for each set, and error bars (the standard error of the mean (SEM)), which is typically less than ± 10% the mean.

Supplemental figure S1

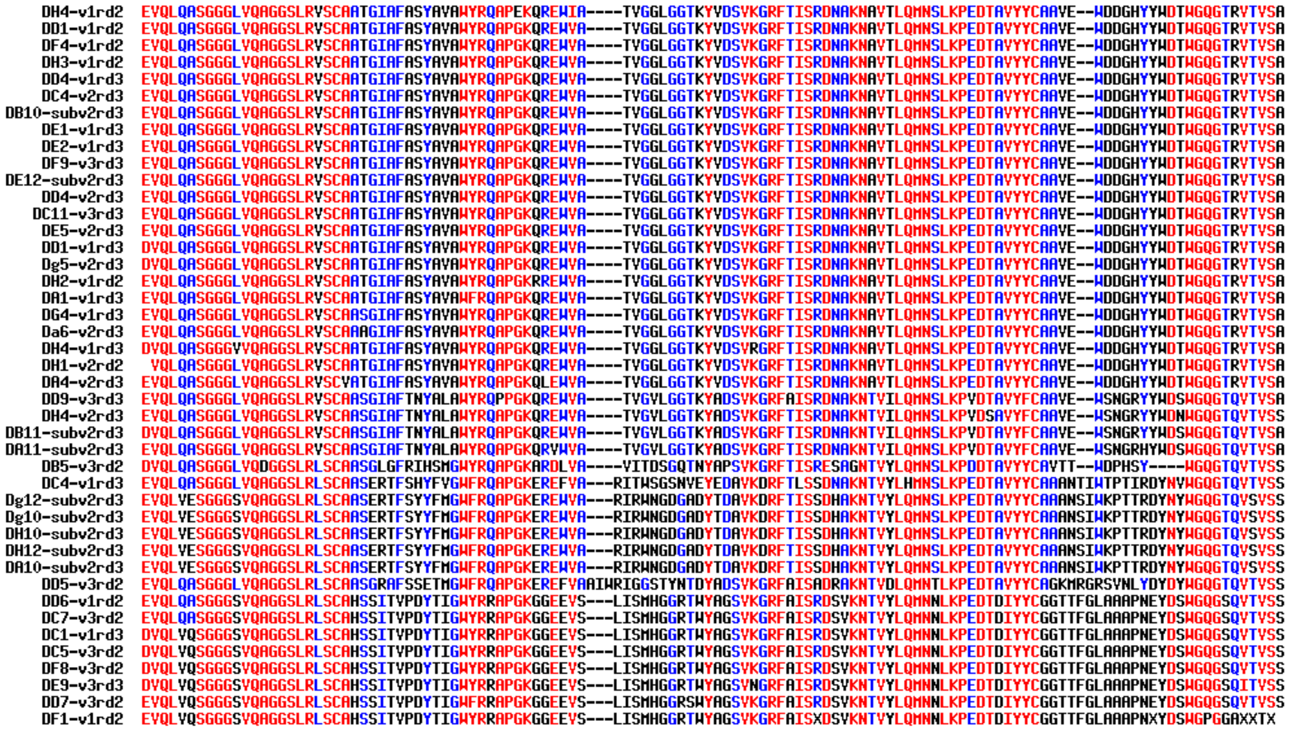

Sequences of clones selected on NS1 from DENV-1, 2, and 3 from second and third rounds. Selection on DENV-1 NS1, round 2 are labeled with “-v1rd2” after the clone name. We identified 10 positives out of 48 screened, and sequenced 7. Round 3 panned on NS1 from DENV-1 are labeled with “v1rd3”. 16 positive clones were identified after screening 32, and 9 were sequenced. Selections on DENV-2 are labeled v2 with the round number; rd2 and rd3 for rounds 2 and 3 respectively. Subtractive panning is noted by the addition of “sub”. In round 2, 3 positives were identified from the regular panning protocol of 24 screened and one was sequenced, while no positives were seen for round 2 of the subtractive panning. For round 3 on NS1 from DENV-2 we identified 11 positives out of 24 screened and sequenced 7. For the subtractive panning we identified 10 binding clones out of 24 screened and sequenced 9. The second round of panning on NS1 from DENV-3 yielded 6 positives out of 34 examined, all were sequenced. The third round gave 5 positives out of 32 and 4 were sequenced.

Sequences fall into 5 sequence families grouped by similarity.

Supplemental figure S2. Direct binding of Bt-sdAb to MagPlex bead immobilized NS1. Control bead sets were included in each experiment. CHIKV VLP indicates beads functionalized with chikungunya virus (CHIKV) virus like particles (VLP). CHIKV VLPs were from the Native Antigen Company (Kidlington, UK)

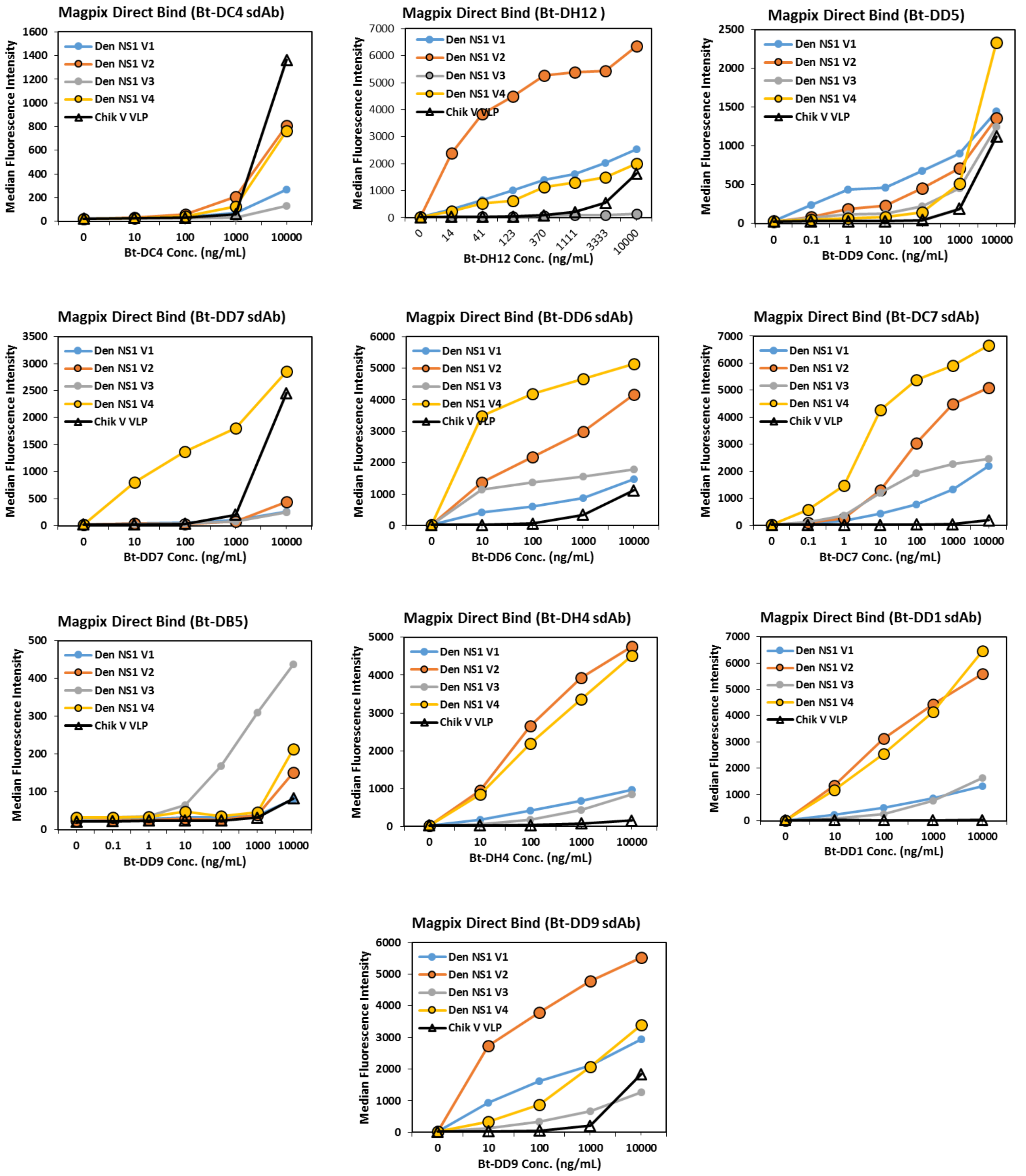

Supplemental figure S3

```
          1       10        20        30        40        50        60        70        80        90        100       110       120       130
          |--------+---------+---------+---------+---------+---------+---------+---------+---------+---------+---------+---------+---------|
DENV-1    DSGCVINWKGRELKCGSGIFVTNEVHTWTEQYKFQADSPKRLSAAIGKAWEEGVCGIRSATRLENIMWKQISNELNHILLENDMKFTVVVGDVSGILAQGKKMIRPQPMEHKYSWKSWGKAKIIGADVQN
DENV-3    DMGCVINWKGKELKCGSGIFVTNEVHTWTEQYKFQADSPKRLATAIAGAWENGVCGIRSTTRMENLLWKQIANELNYILWENNIKLTVVVGDTLGVLEQGKRTLTPQPMELKYSWKTWGKAKIVTAETQN
DENV-2    DSGCVVSWKNKELKCGSGIFITDNVHTWTEQYKFQPESPSKLASAIQKAHEEGICGIRSVTRLENLMWKQITPELNHILSENEVKLTIMTGDIKGIMQAGKRSLRPQPTELKYSWKTWGKAKMLSTESHN
DENV-4    DMGCVASWSGKELKCGSGIFVVDNVHTWTEQYKFQPESPARLASAILNAHKDGVCGIRSTTRLENVMWKQITNELNYVLWEGGHDLTVVAGDVKGVLTKGKRALTPPVSDLKYSWKTWGKAKIFTPEARN
Consensus DmGCV.sWkgkELKCGSGIF!t##VHTWTEQYKFQp#SP.rLasAI..Ahe#G!CGIRStTR$EN.$WKQItnELNy!LwEn..klT!v.GDvkG!$..GKr.ltPqp.#lKYSWKtWGKAKi.t.#..N

          131     140       150       160       170       180       190       200       210       220       230       240       250       260
          |--------+---------+---------+---------+---------+---------+---------+---------+---------+---------+---------+---------+---------|
DENV-1    TTFIIDGPNTPECPDNQRAWNIWEVEDYGFGIFTTNIWLKLRDSYTQVCDHRLMSAAIKDSKAVHADMGYWIESEKNETWKLARASFIEVKTCIWPKSHTLWSNGVLESEMIIPKIYGGPISQHNYRPGY
DENV-3    SSFIIDGPNTPECPSASRAWNVWEVEDYGFGVFTTNIWLKLREVYTQLCDHRLMSAAVKDERAVHADMGYWIESQKNGSWKLEKASLIEVKTCTWPKSHTLWTNGVLESDMIIPKSLAGPISQHNYRPGY
DENV-2    QTFLIDGPETAECPNTNRAWNSLEVEDYGFGVFTTNIWLKLKEKQDVFCDSKLMSAAIKDNRAVHADMGYWIESALNDTWKIEKASFIEVKNCHWPKSHTLWSNGVLESEMIIPKNLAGPVSQHNYRPGY
DENV-4    STFLIDGPDTSECPNERRAWNSLEVEDYGFGMFTTNIWMKFREGSSEVCDHRLMSAAIKDQKAVHADMGYWIESSKNQTWQIEKASLIEVKTCLWPKTHTLWSNGVLESQMLIPKSYAGPFSQHNYRQGY
Consensus stFlIDGP#T.ECPn..RAWNslEVEDYGFG.FTTNIW$Klr#....vCDhrLMSAA!KD.kAVHADMGYWIES.kN.tWkiekASlIEVKtC.WPKsHTLWsNGVLES#MiIPKsyaGP.SQHNYRpGY

          261     270       280       290       300       310       320       330       340       35052
          |--------+---------+---------+---------+---------+---------+---------+---------+---------+-|
DENV-1    FTQTAGPWHLGKLELDFDLCEGTTVVVDEHCGNRGPSLRTTTVTGKTIHEWCCRSCTLPPLRFKGEDGCWYGMEIRPVKEKEENLVKSMVSA
DENV-3    HTQTAGPWHLGKLELDFNYCEGTTVVITESCGTRGPSLRTTTVSGKLIHEWCCRSCTLPPLRYMGEDGCWYGMEIRPISEKEENMVKSLVSA
DENV-2    HTQITGPWHLGKLEMDFDFCDGTTVVVTEDCGNRGPSLRTTTASGKLITEWCCRSCTLPPLRYRGEDGCWYGMEIRPLKEKEENLVNSLVTA
DENV-4    ATQTVGPWHLGKLEIDFGECPGTTVTIQEDCDHRGPSLRTTTASGKLVTQWCCRSCTMPPLRFLGEDGCWYGMEIRPLSEKEENMVKSQVTA
Consensus .TQt.GPWHLGKLE.DF..C.GTTVv!.EdCg.RGPSLRTTTasGKl!t#WCCRSCT$PPLR%.GEDGCWYGMEIRPlsEKEEN$VkS.VtA
```

Comparison of the primary sequence of the DENV-1-4 NS1 strains Nauru/West Pac/1974; Thailand/16681/1984; Sri Lanka/1266/2000; Dominica/814669/1981 respectively. These are the strains from which the recombinant NS1 from The Native Antigen Company are derived.

Supplemental figure S4. Surface Plasmon Resonance Data for SdAb. The NS1 from DENV-1, DENV-2, DENV-3 and DENV-4 are noted as V1, V2, V3, and V4 respectively. Concentrations indicated in nM to the right of each set of traces. DC4 and DH12 are also shown scaled independently after all the other sdAb for better visualization.

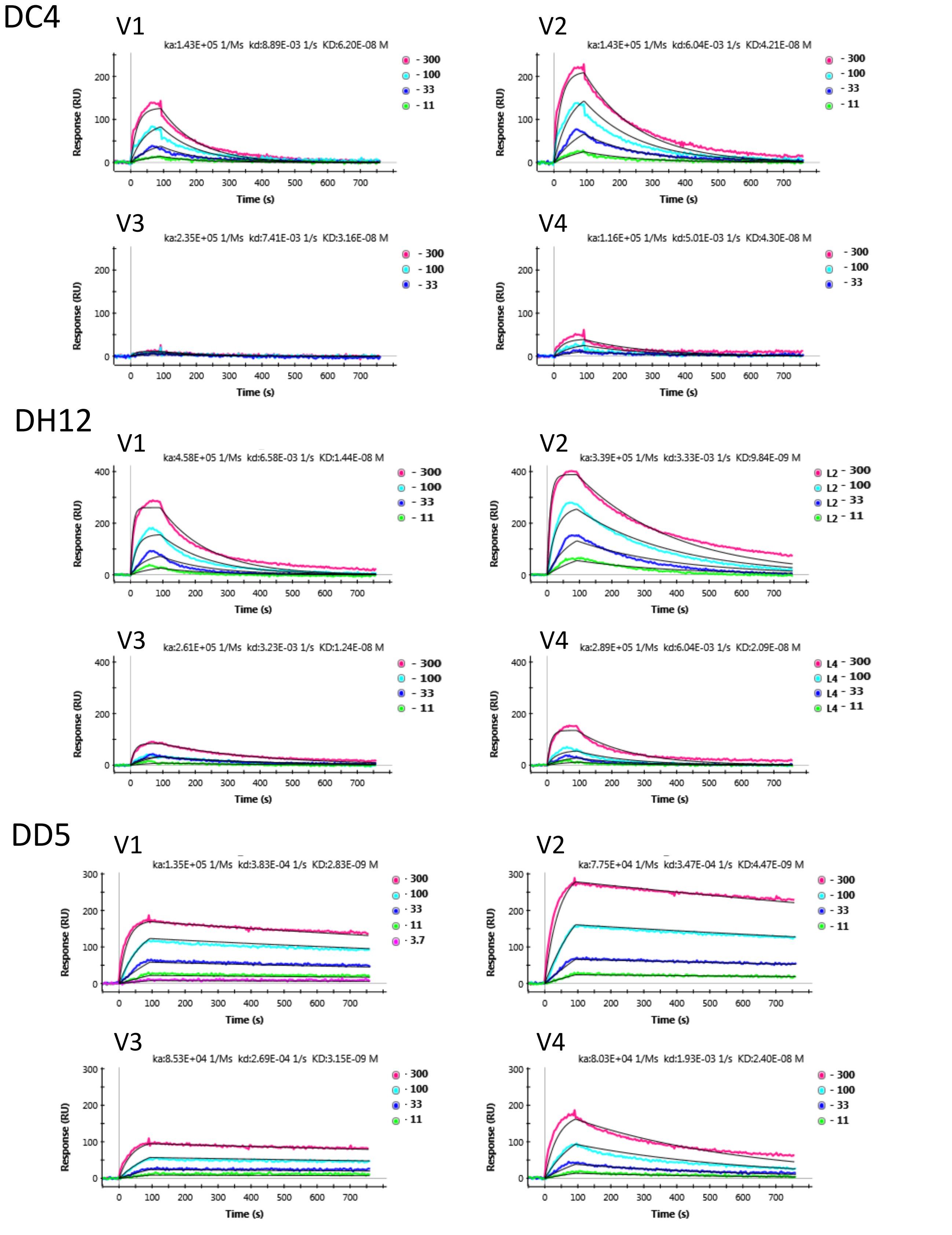

DD7

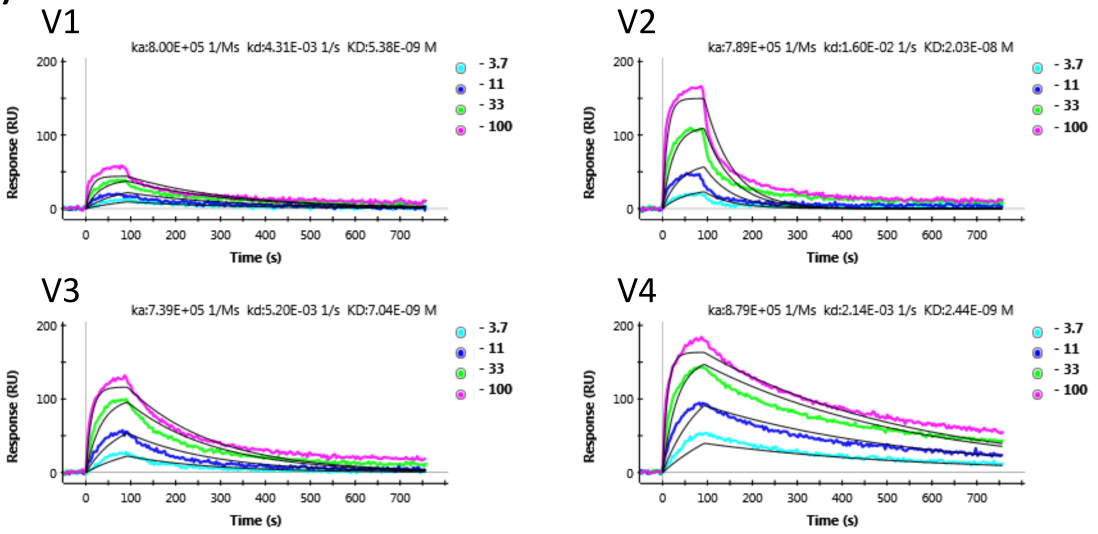
V1
ka:8.00E+05 1/Ms kd:4.31E-03 1/s KD:5.38E-09 M
- 3.7
- 11
- 33
- 100
V2
ka:7.89E+05 1/Ms kd:1.60E-02 1/s KD:2.03E-08 M
- 3.7
- 11
- 33
- 100
V3
ka:7.39E+05 1/Ms kd:5.20E-03 1/s KD:7.04E-09 M
- 3.7
- 11
- 33
- 100
V4
ka:8.79E+05 1/Ms kd:2.14E-03 1/s KD:2.44E-09 M
- 3.7
- 11
- 33
- 100
Response (RU)
Time (s)

DD6

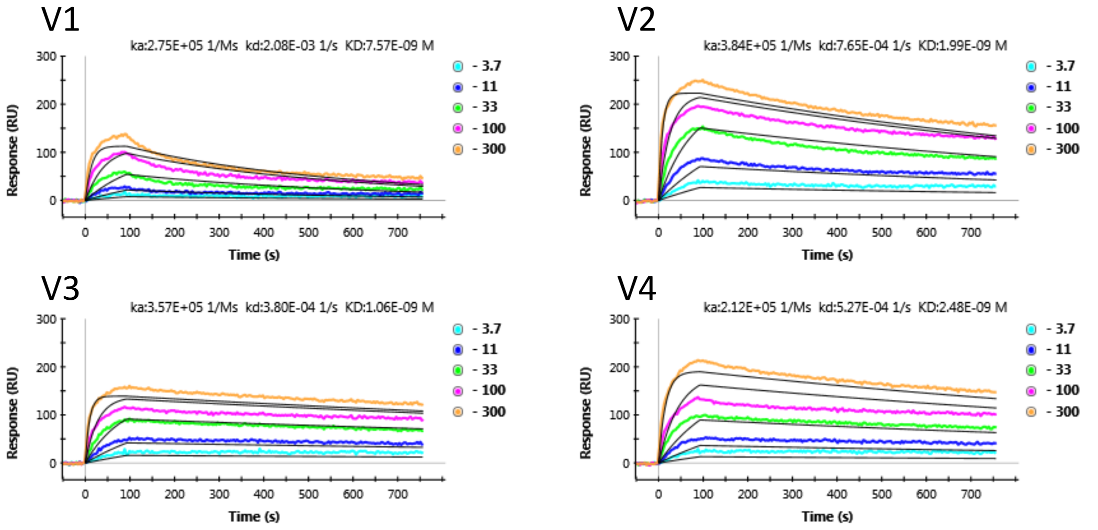
V1
ka:2.75E+05 1/Ms kd:2.08E-03 1/s KD:7.57E-09 M
- 3.7
- 11
- 33
- 100
- 300
V2
ka:3.84E+05 1/Ms kd:7.65E-04 1/s KD:1.99E-09 M
- 3.7
- 11
- 33
- 100
- 300
V3
ka:3.57E+05 1/Ms kd:3.80E-04 1/s KD:1.06E-09 M
- 3.7
- 11
- 33
- 100
- 300
V4
ka:2.12E+05 1/Ms kd:5.27E-04 1/s KD:2.48E-09 M
- 3.7
- 11
- 33
- 100
- 300
Response (RU)
Time (s)

DC7

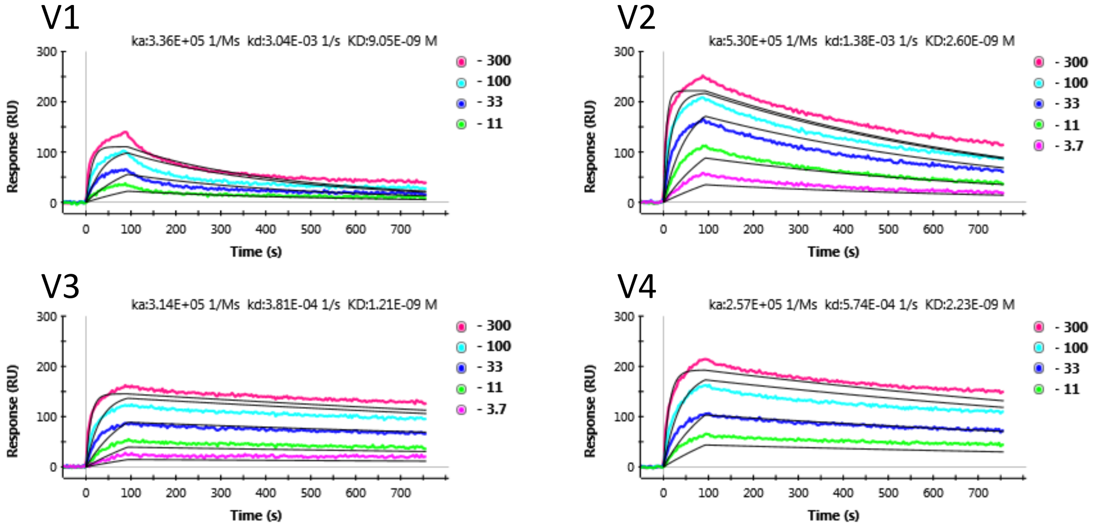
V1
ka:3.36E+05 1/Ms kd:3.04E-03 1/s KD:9.05E-09 M
- 300
- 100
- 33
- 11
V2
ka:5.30E+05 1/Ms kd:1.38E-03 1/s KD:2.60E-09 M
- 300
- 100
- 33
- 11
- 3.7
V3
ka:3.14E+05 1/Ms kd:3.81E-04 1/s KD:1.21E-09 M
- 300
- 100
- 33
- 11
- 3.7
V4
ka:2.57E+05 1/Ms kd:5.74E-04 1/s KD:2.23E-09 M
- 300
- 100
- 33
- 11
Response (RU)
Time (s)

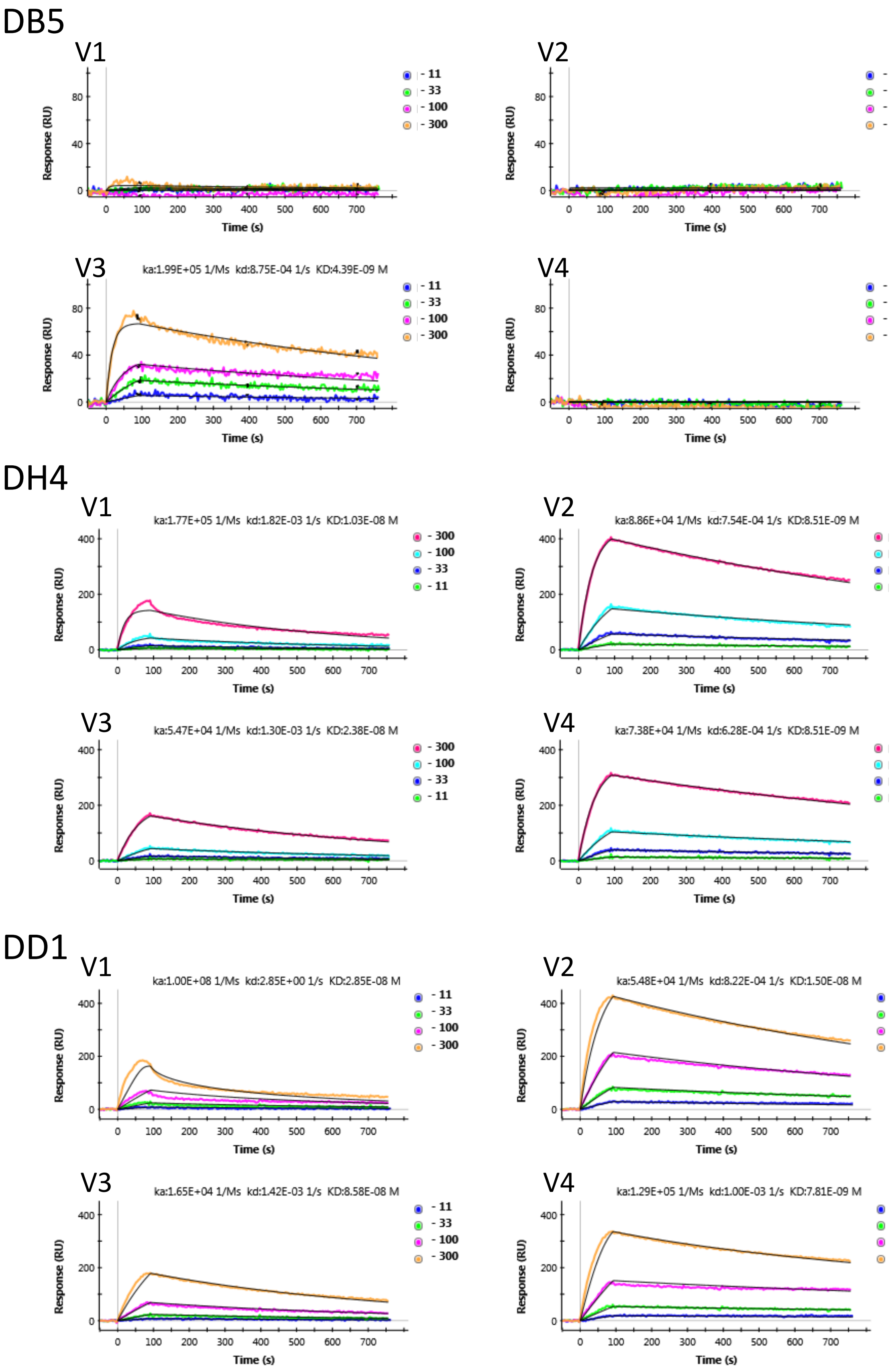
DB5
V1
V2
V3
ka:1.99E+05 1/Ms kd:8.75E-04 1/s KD:4.39E-09 M
V4
DH4
V1
ka:1.77E+05 1/Ms kd:1.82E-03 1/s KD:1.03E-08 M
V2
ka:8.86E+04 1/Ms kd:7.54E-04 1/s KD:8.51E-09 M
V3
ka:5.47E+04 1/Ms kd:1.30E-03 1/s KD:2.38E-08 M
V4
ka:7.38E+04 1/Ms kd:6.28E-04 1/s KD:8.51E-09 M
DD1
V1
ka:1.00E+08 1/Ms kd:2.85E+00 1/s KD:2.85E-08 M
V2
ka:5.48E+04 1/Ms kd:8.22E-04 1/s KD:1.50E-08 M
V3
ka:1.65E+04 1/Ms kd:1.42E-03 1/s KD:8.58E-08 M
V4
ka:1.29E+05 1/Ms kd:1.00E-03 1/s KD:7.81E-09 M
Response (RU)
Time (s)
- 11
- 33
- 100
- 300

DD9

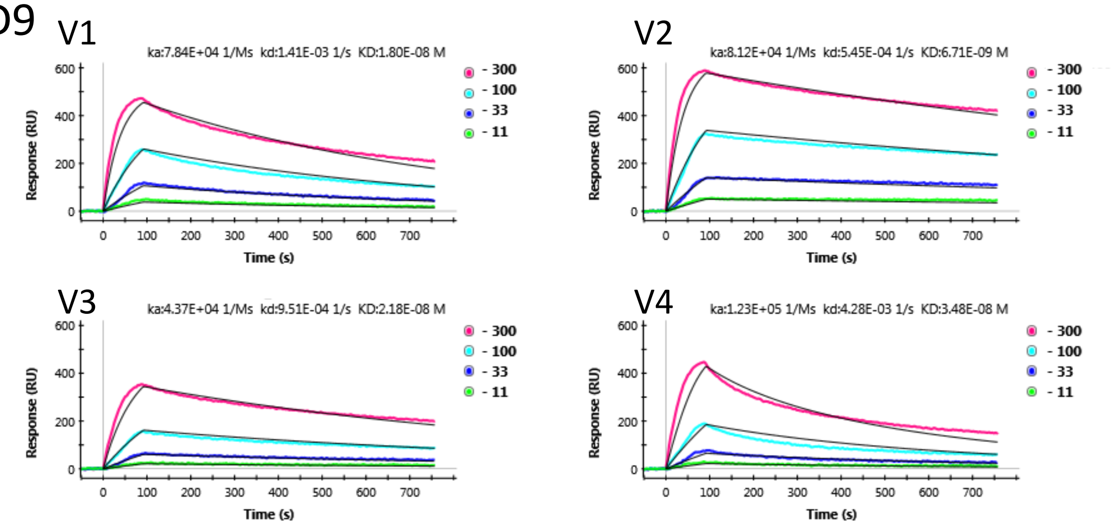

DC4-scaled independently

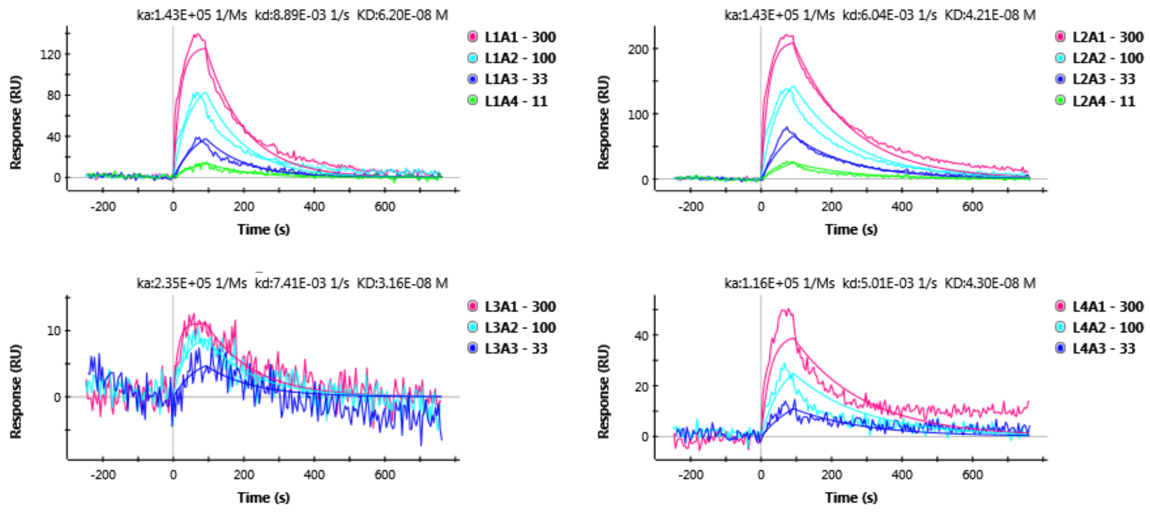

DH12-scaled independently

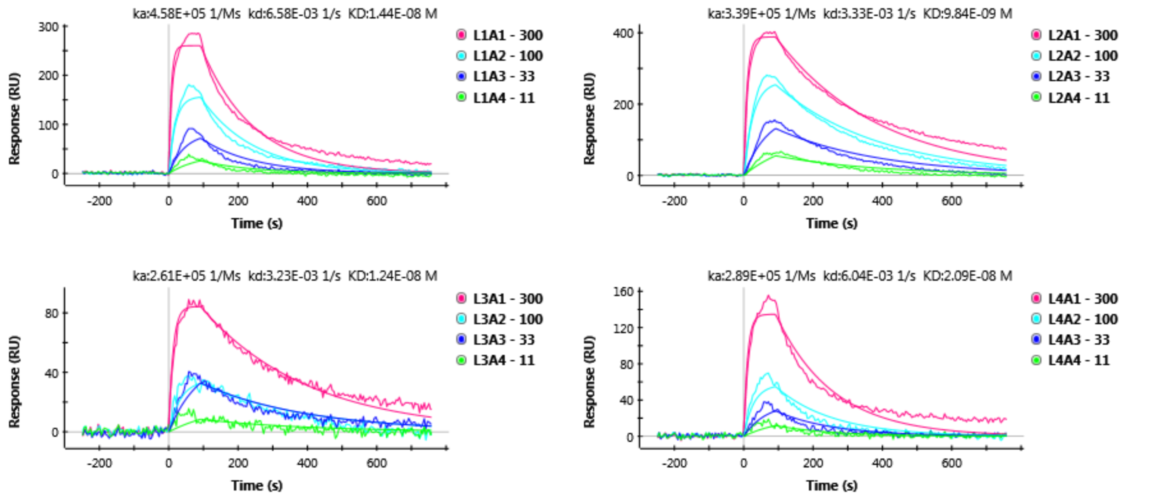

Supplemental figure S5. SPR data for immobilized sdAb binding NS1. The NS1 from DENV-1, DENV-2, DENV-3 and DENV-4 are noted as V1, V2, V3, and V4 respectively. Concentrations indicated in nM to the right of each set of traces.

V1

DD1

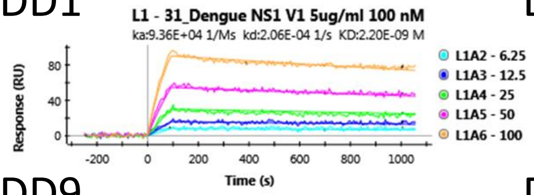

DD7

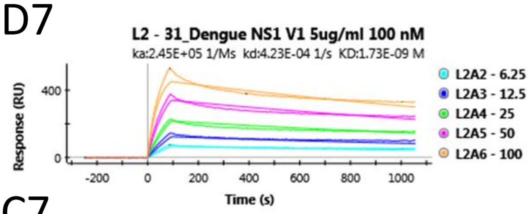

DD9

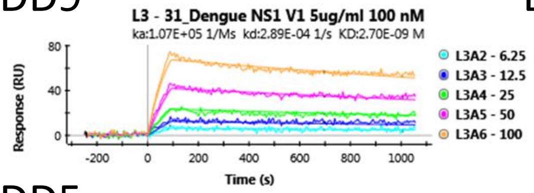

DC7

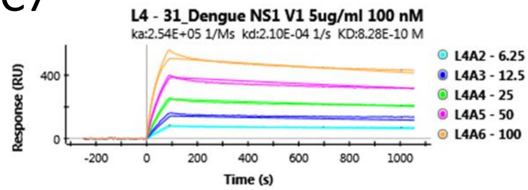

DD5

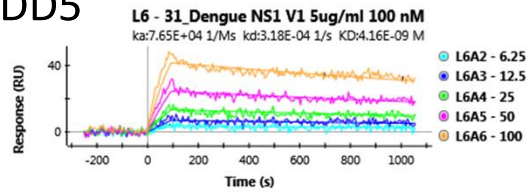

V2

DD1

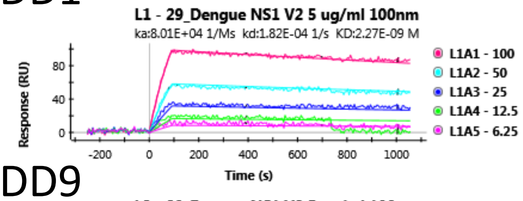

DD7

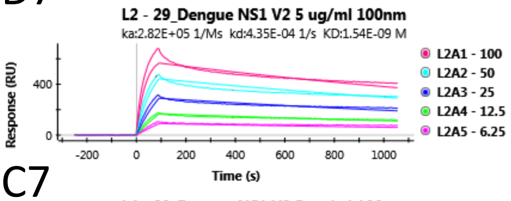

DD9

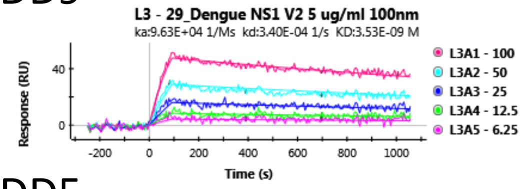

DC7

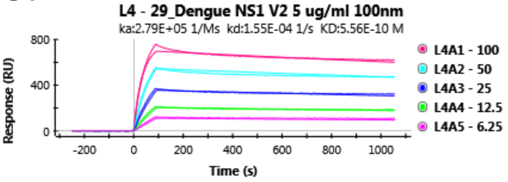

DD5

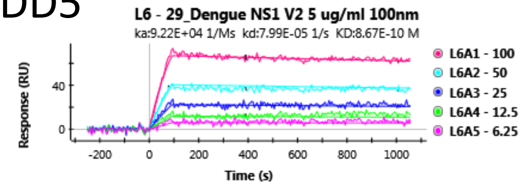

V3

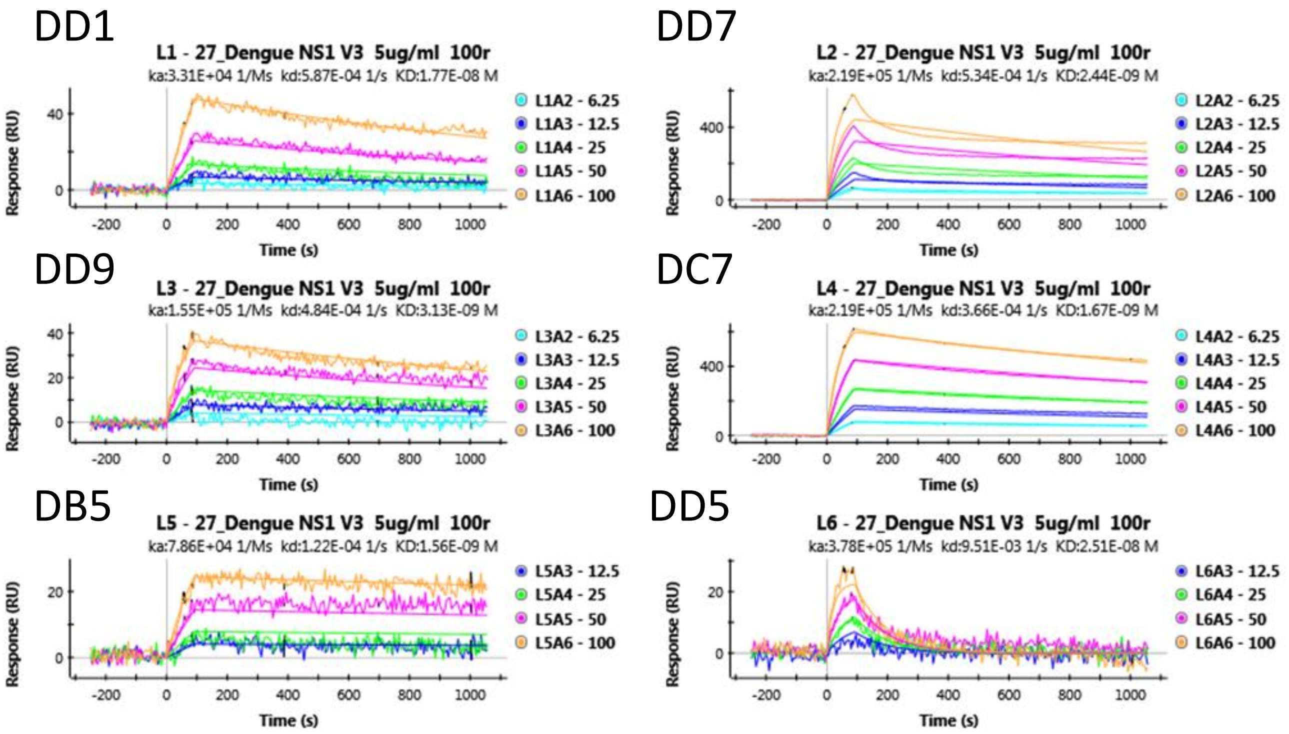
DD1
L1 - 27_Dengue NS1 V3 5ug/ml 100r
ka:3.31E+04 1/Ms kd:5.87E-04 1/s KD:1.77E-08 M
L1A2 - 6.25
L1A3 - 12.5
L1A4 - 25
L1A5 - 50
L1A6 - 100
DD7
L2 - 27_Dengue NS1 V3 5ug/ml 100r
ka:2.19E+05 1/Ms kd:5.34E-04 1/s KD:2.44E-09 M
L2A2 - 6.25
L2A3 - 12.5
L2A4 - 25
L2A5 - 50
L2A6 - 100
DD9
L3 - 27_Dengue NS1 V3 5ug/ml 100r
ka:1.55E+05 1/Ms kd:4.84E-04 1/s KD:3.13E-09 M
L3A2 - 6.25
L3A3 - 12.5
L3A4 - 25
L3A5 - 50
L3A6 - 100
DC7
L4 - 27_Dengue NS1 V3 5ug/ml 100r
ka:2.19E+05 1/Ms kd:3.66E-04 1/s KD:1.67E-09 M
L4A2 - 6.25
L4A3 - 12.5
L4A4 - 25
L4A5 - 50
L4A6 - 100
DB5
L5 - 27_Dengue NS1 V3 5ug/ml 100r
ka:7.86E+04 1/Ms kd:1.22E-04 1/s KD:1.56E-09 M
L5A3 - 12.5
L5A4 - 25
L5A5 - 50
L5A6 - 100
DD5
L6 - 27_Dengue NS1 V3 5ug/ml 100r
ka:3.78E+05 1/Ms kd:9.51E-03 1/s KD:2.51E-08 M
L6A3 - 12.5
L6A4 - 25
L6A5 - 50
L6A6 - 100
Response (RU)
Time (s)

V4

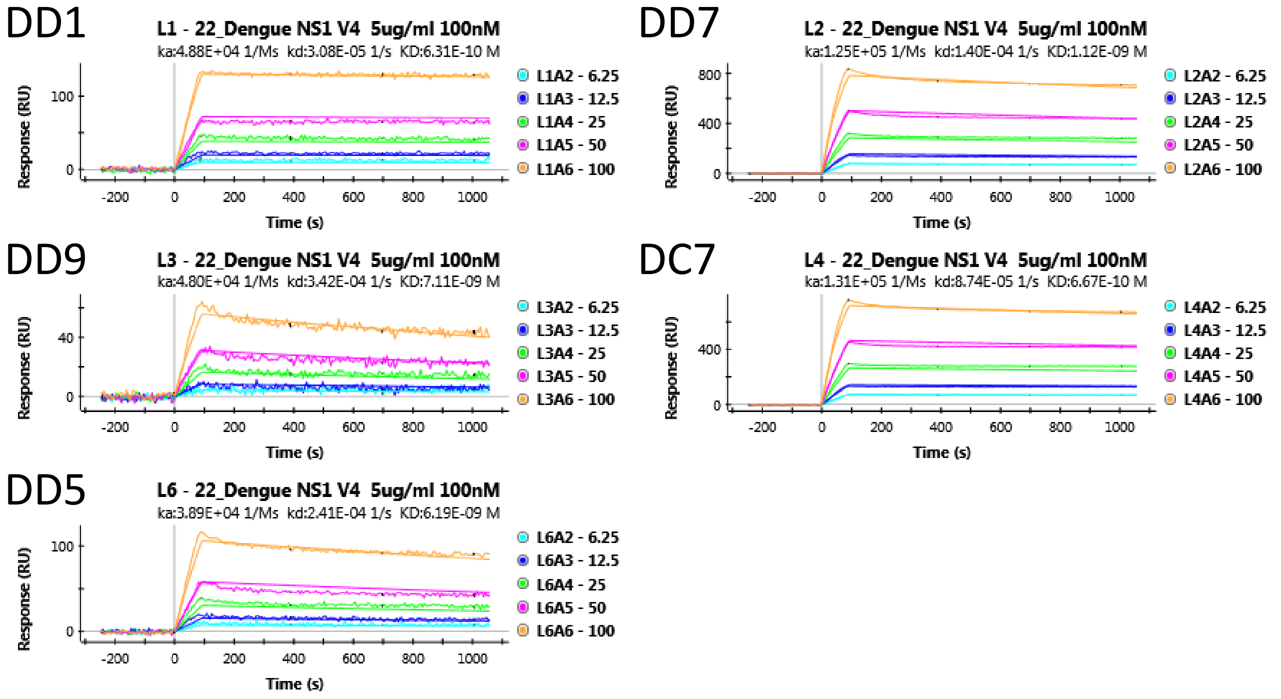
DD1
L1 - 22_Dengue NS1 V4 5ug/ml 100nM
ka:4.88E+04 1/Ms kd:3.08E-05 1/s KD:6.31E-10 M
L1A2 - 6.25
L1A3 - 12.5
L1A4 - 25
L1A5 - 50
L1A6 - 100
DD7
L2 - 22_Dengue NS1 V4 5ug/ml 100nM
ka:1.25E+05 1/Ms kd:1.40E-04 1/s KD:1.12E-09 M
L2A2 - 6.25
L2A3 - 12.5
L2A4 - 25
L2A5 - 50
L2A6 - 100
DD9
L3 - 22_Dengue NS1 V4 5ug/ml 100nM
ka:4.80E+04 1/Ms kd:3.42E-04 1/s KD:7.11E-09 M
L3A2 - 6.25
L3A3 - 12.5
L3A4 - 25
L3A5 - 50
L3A6 - 100
DC7
L4 - 22_Dengue NS1 V4 5ug/ml 100nM
ka:1.31E+05 1/Ms kd:8.74E-05 1/s KD:6.67E-10 M
L4A2 - 6.25
L4A3 - 12.5
L4A4 - 25
L4A5 - 50
L4A6 - 100
DD5
L6 - 22_Dengue NS1 V4 5ug/ml 100nM
ka:3.89E+04 1/Ms kd:2.41E-04 1/s KD:6.19E-09 M
L6A2 - 6.25
L6A3 - 12.5
L6A4 - 25
L6A5 - 50
L6A6 - 100
Response (RU)
Time (s)

Supplemental figure S6a. MagPlex Sandwich Assays for DENV-1 NS1

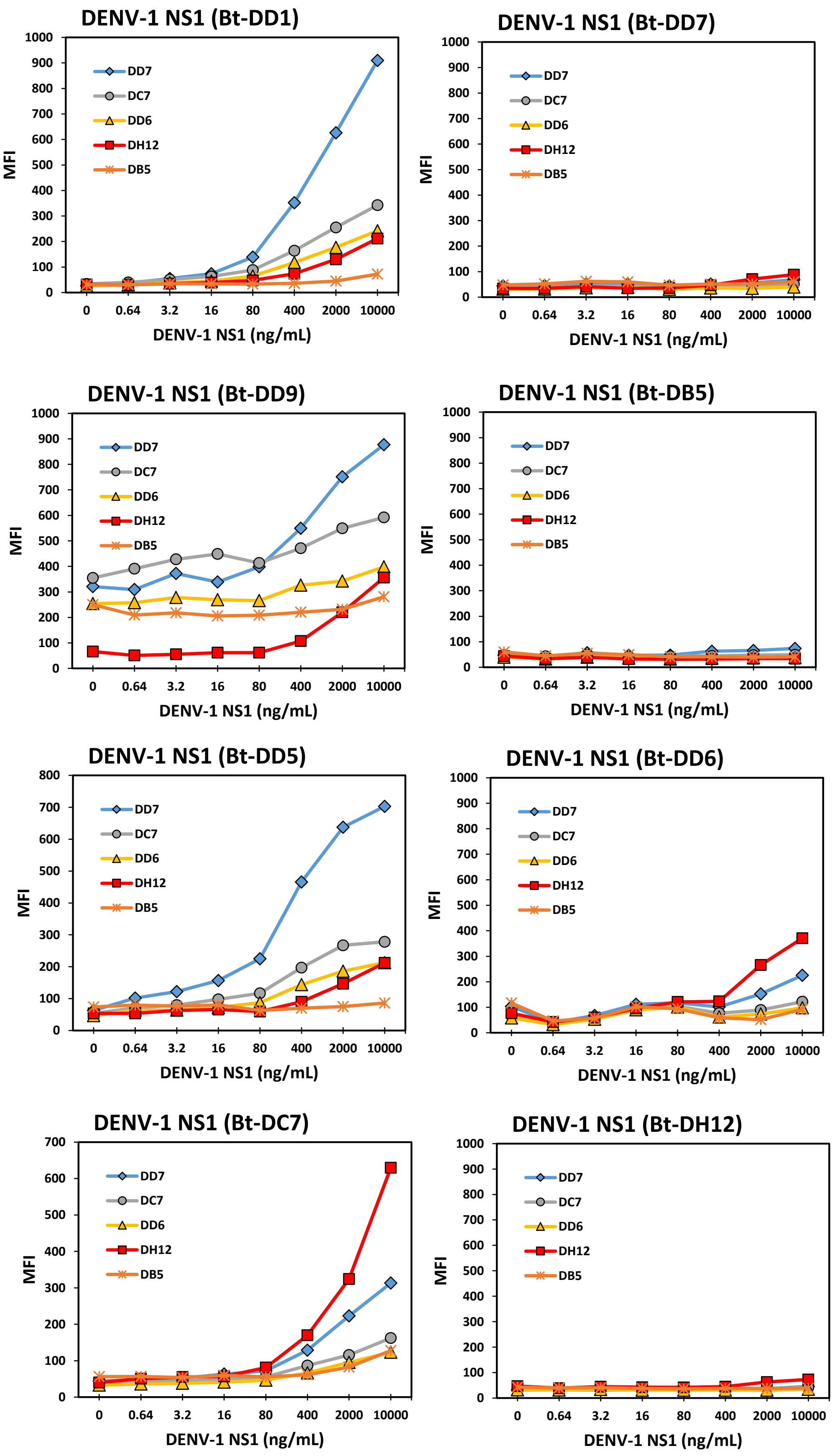

Supplemental figure S6b. MagPlex Sandwich Assays for DENV-2 NS1

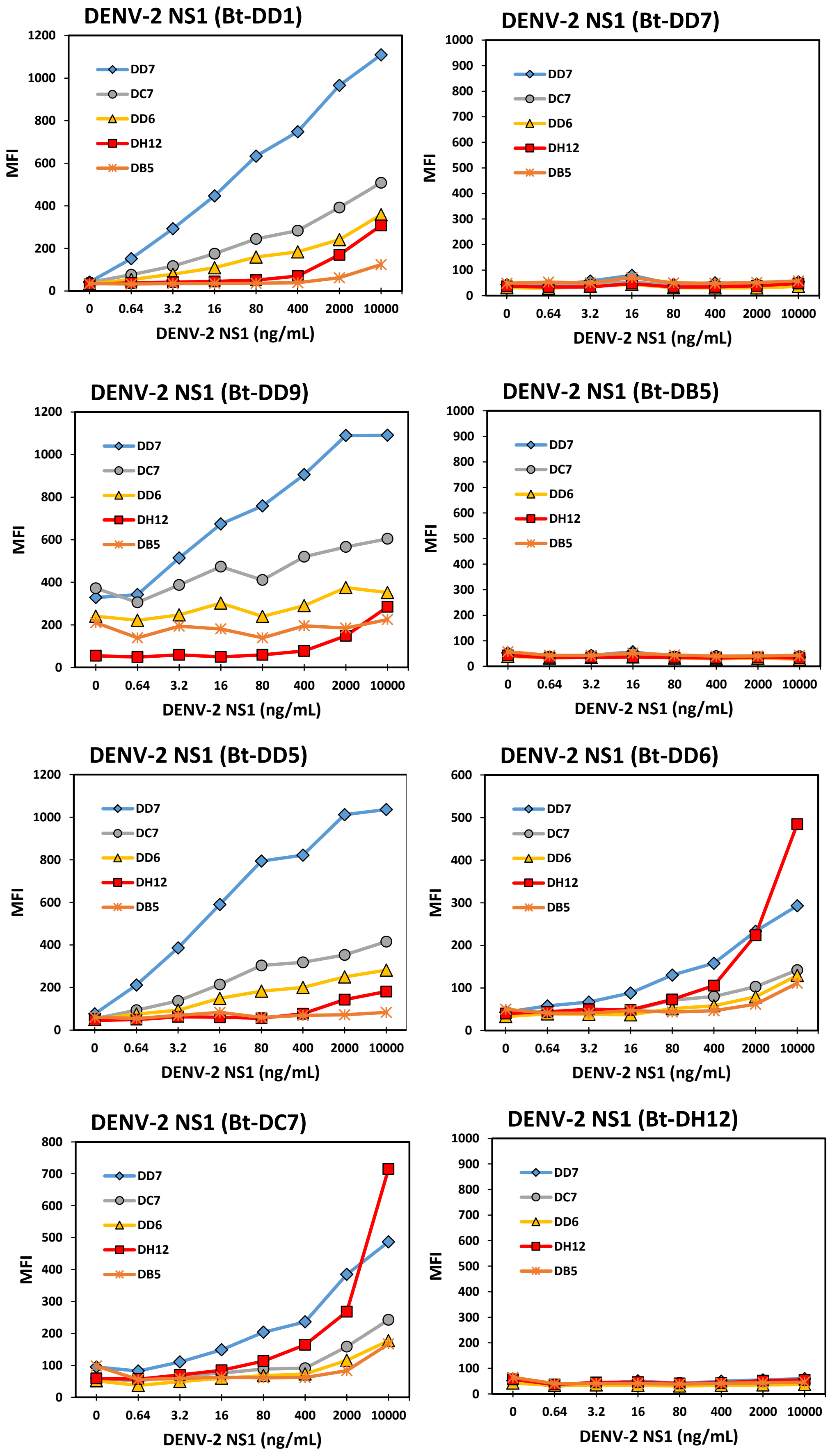

Supplemental figure S6c. MagPlex Sandwich Assays for DENV-3 NS1

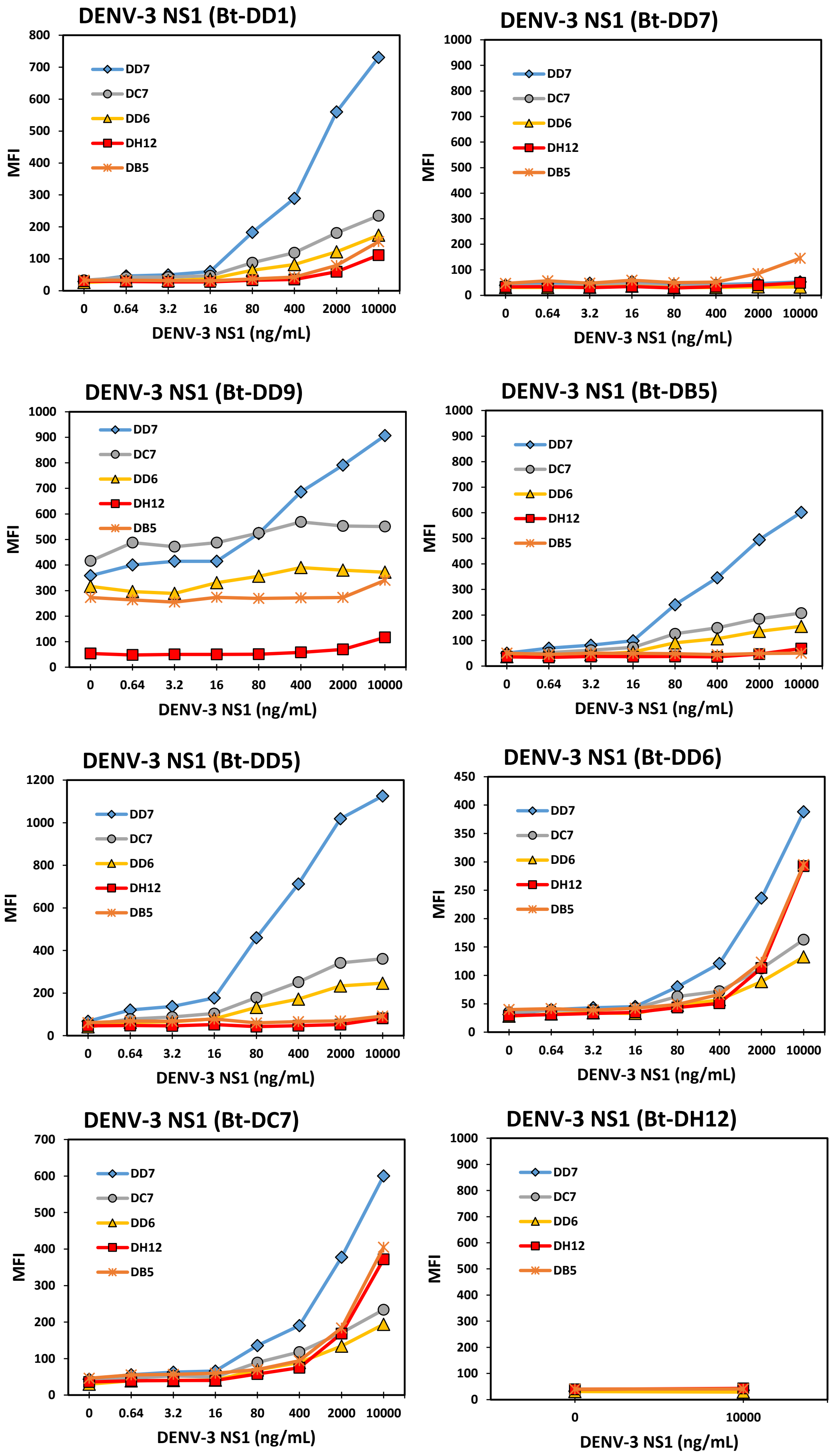

Supplemental figure S6d. MagPlex Sandwich Assays for DENV-4 NS1

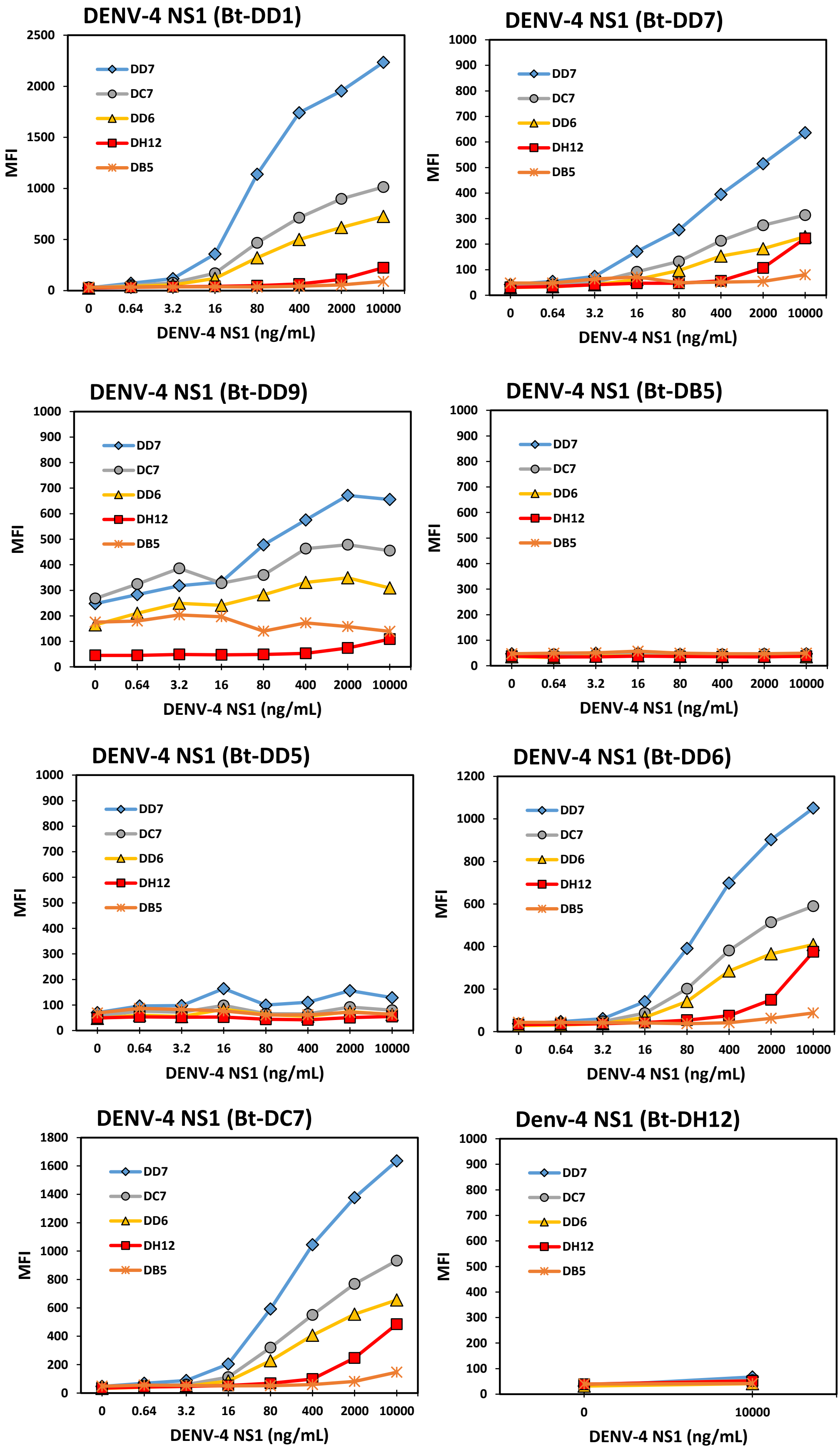

Supplemental figure S7a. MagPlex Sandwich Assays for DENV-1 -4 NS1 (tested as a mixture of serotypes)

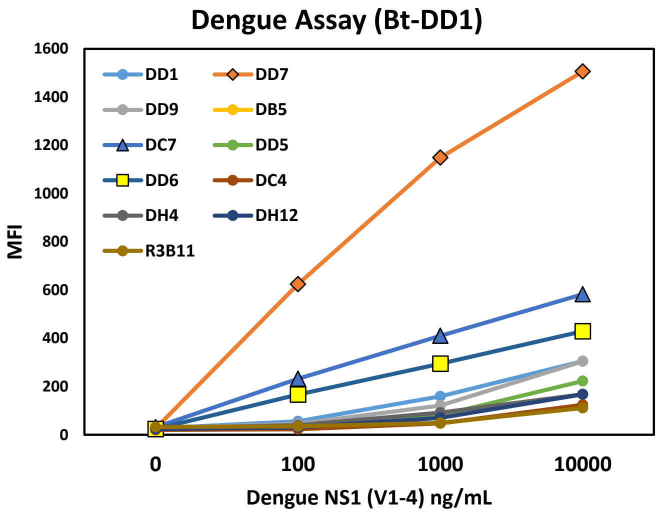

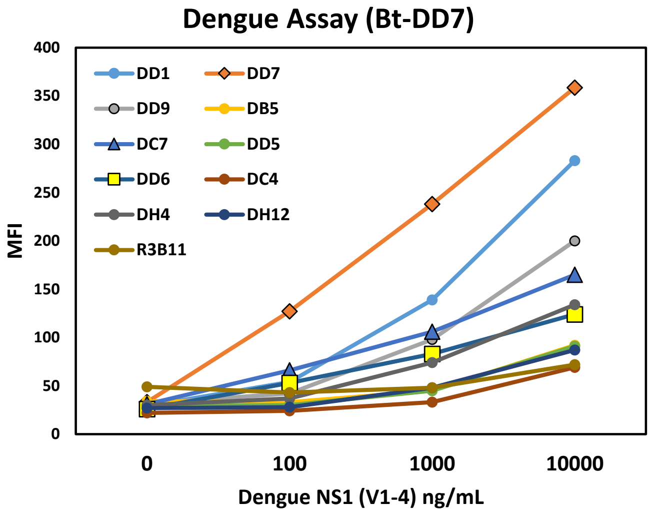

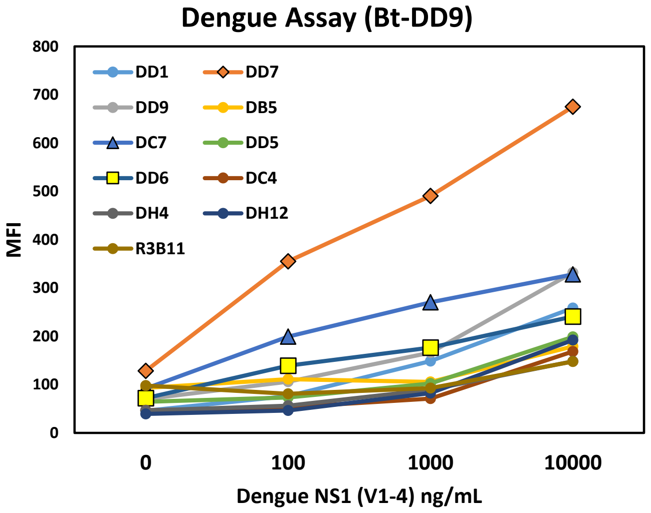

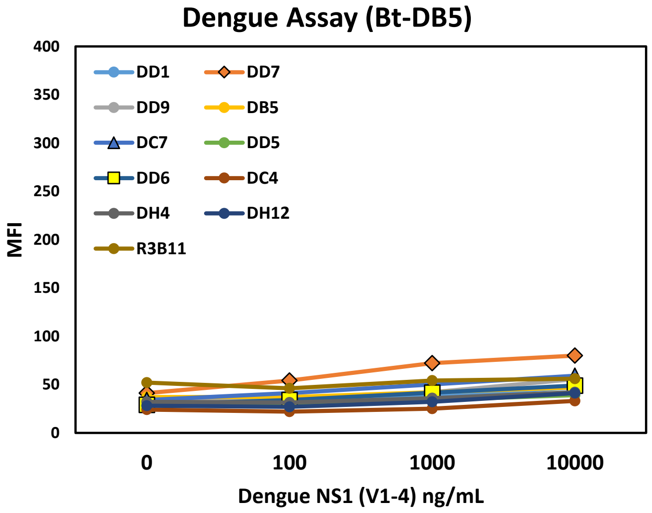

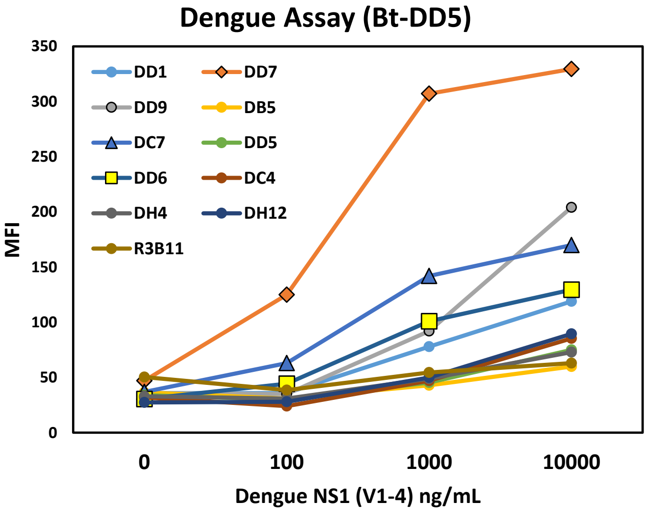

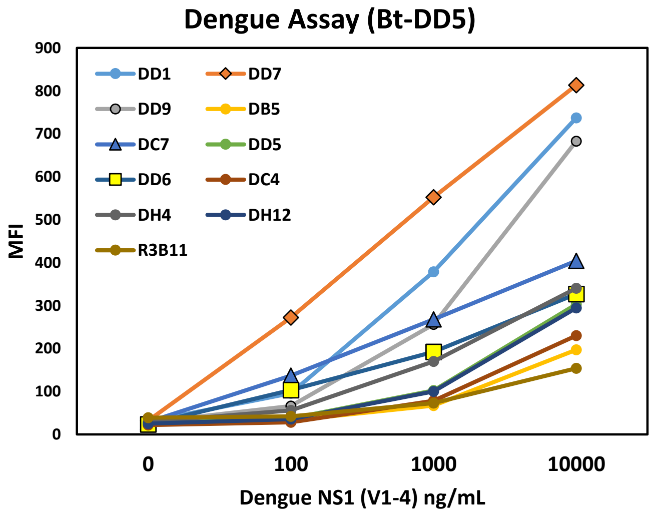

Supplemental Figure S7b. MagPlex Sandwich Assays for ZIKV NS1

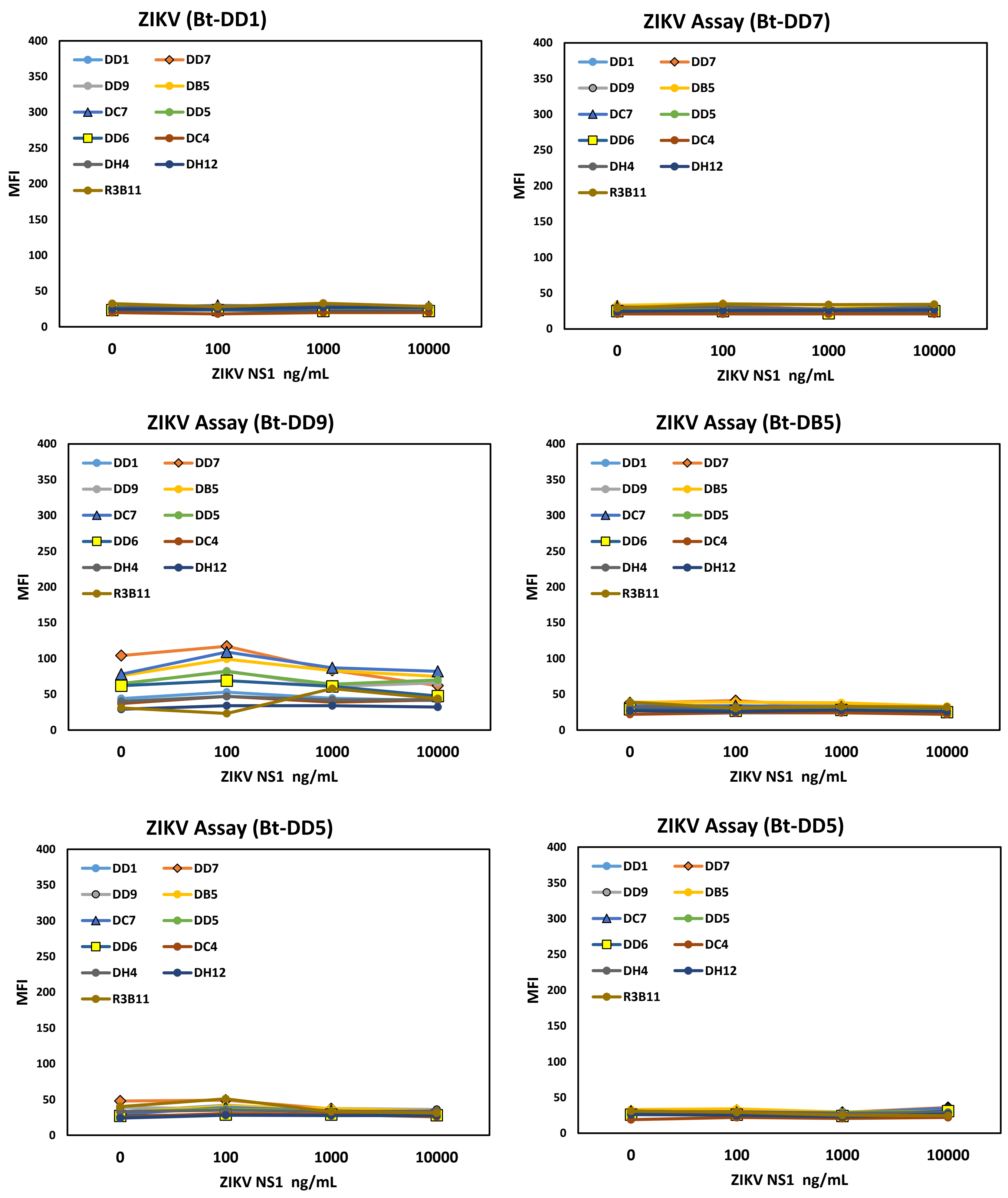

Supplemental figure S7c. MagPlex Sandwich Assays for YFV NS1

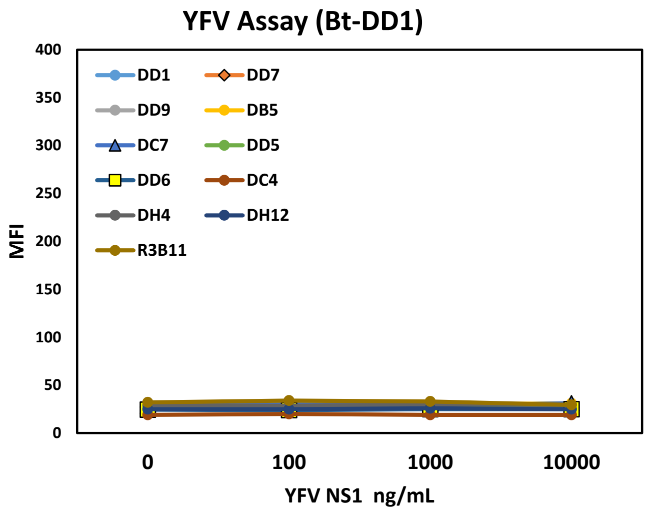

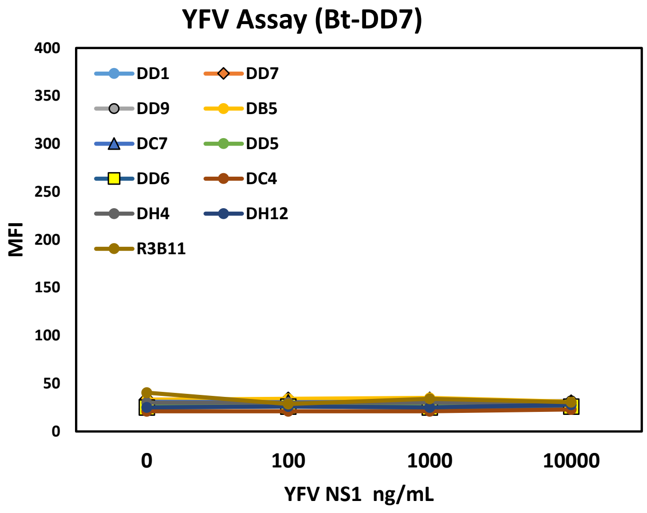

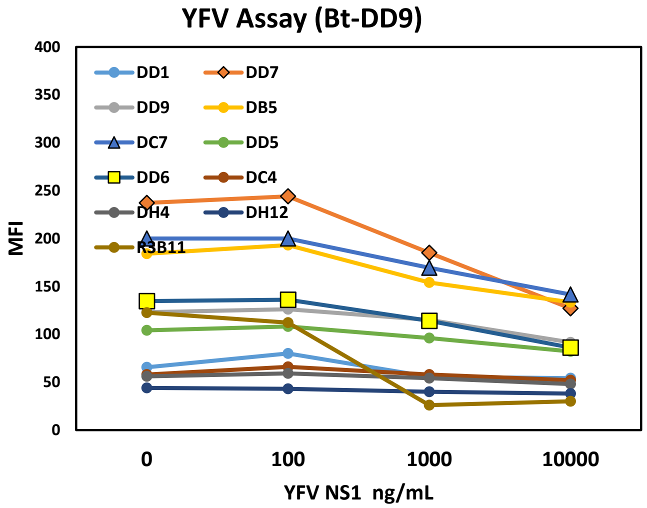

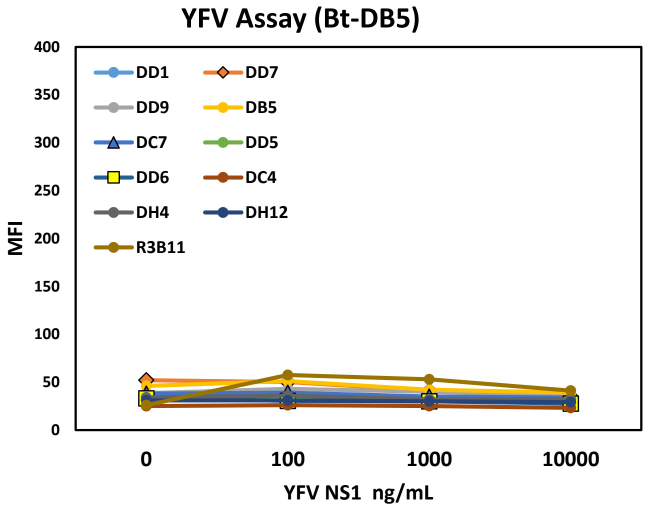

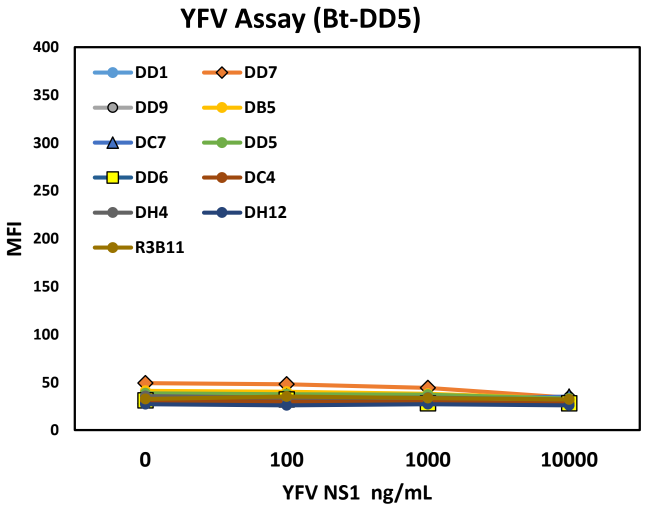

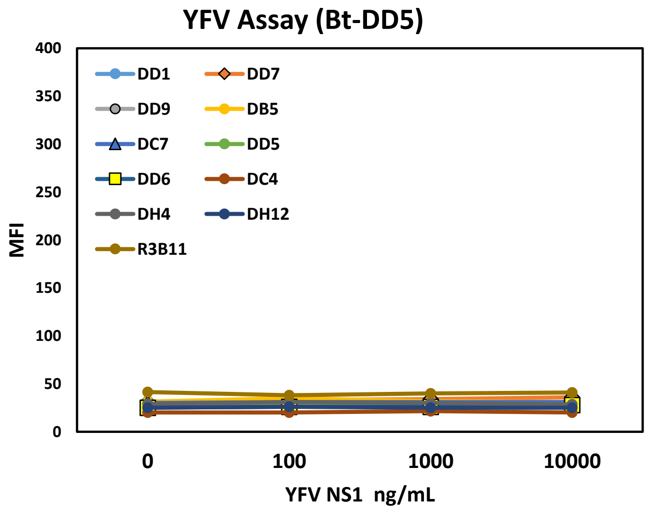

Supplemental figure S7d. MagPlex Sandwich Assays for JEV NS1

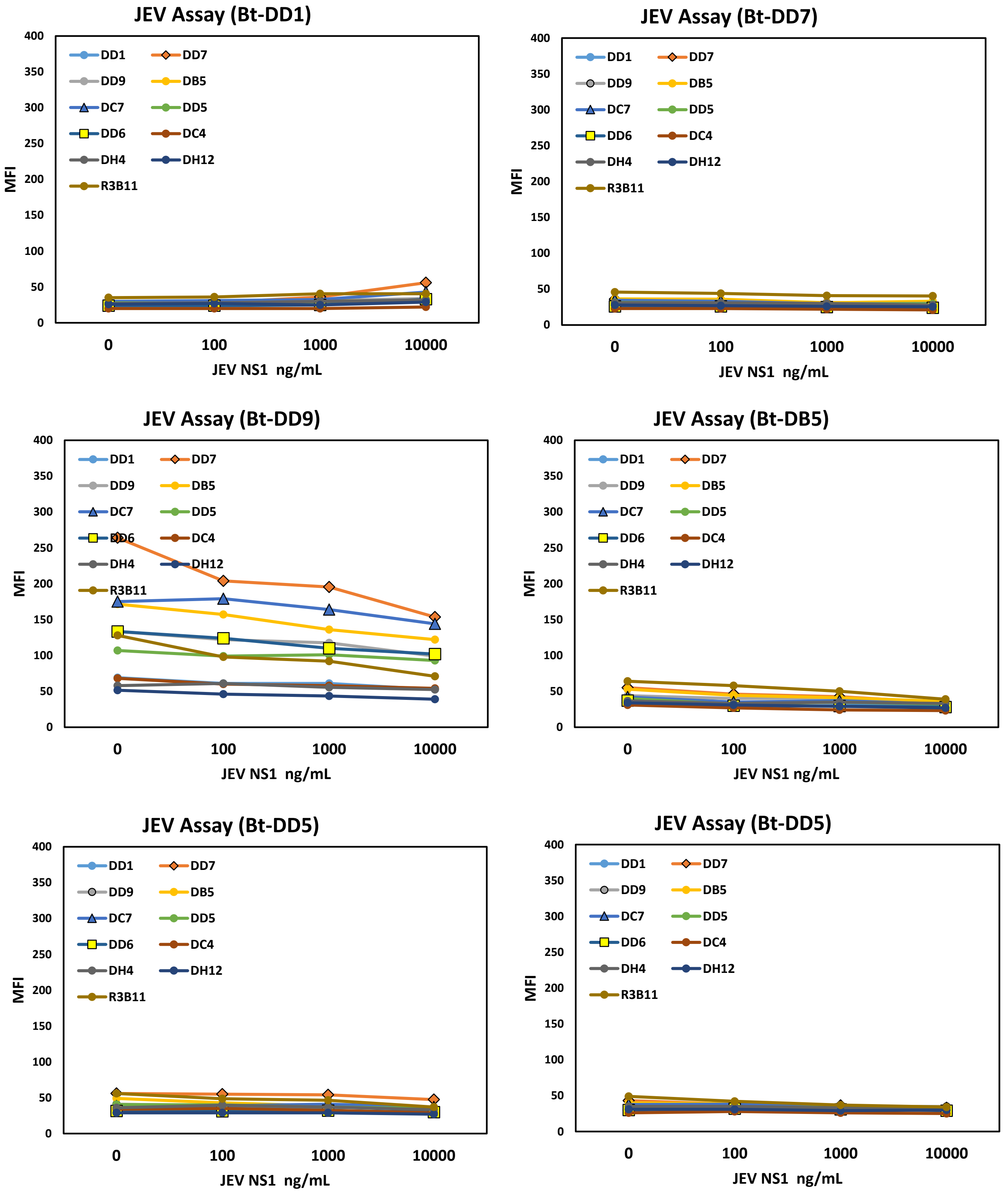

Supplemental figure S7e. MagPlex Sandwich Assays for WNV NS1

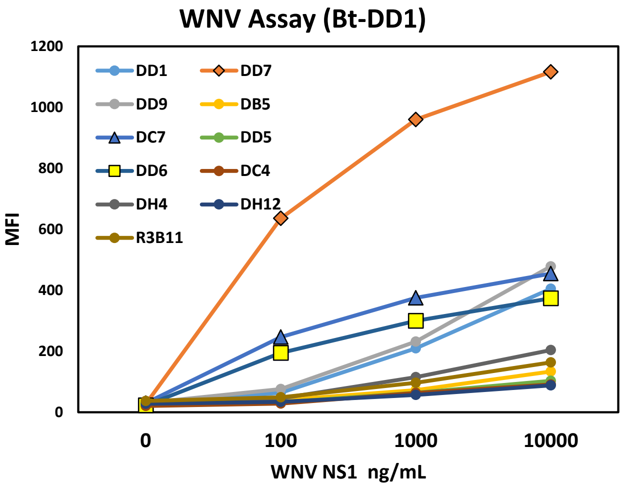

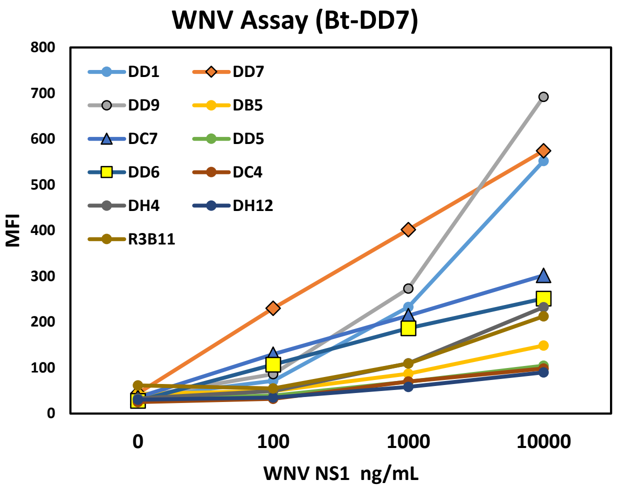

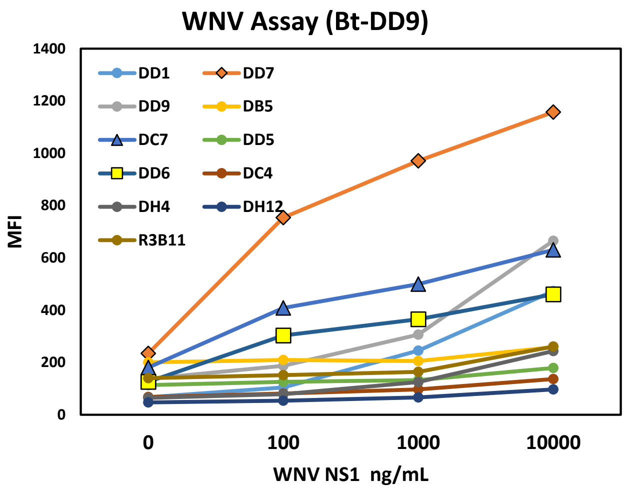

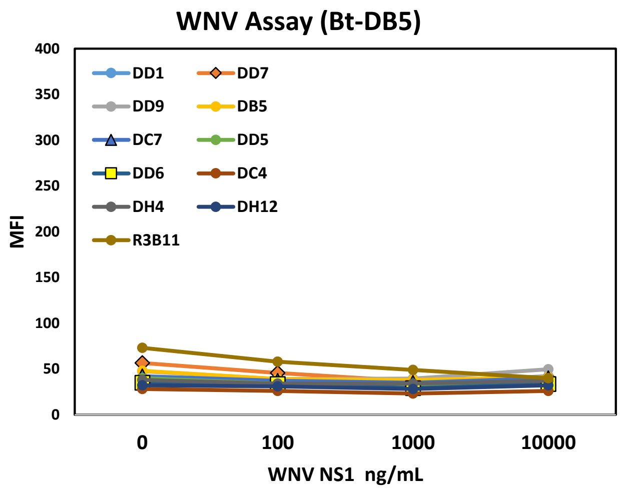

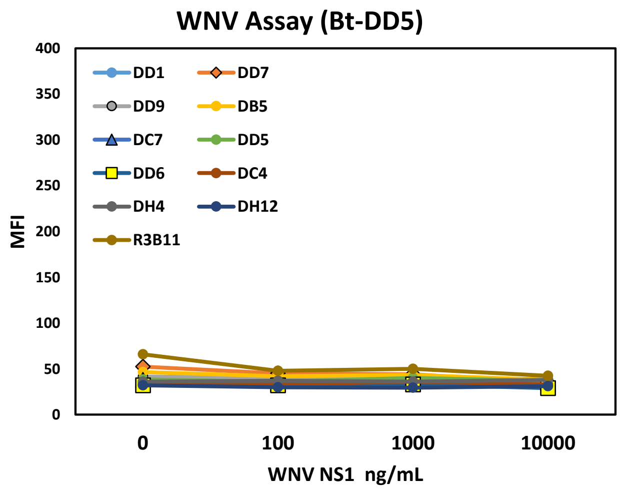

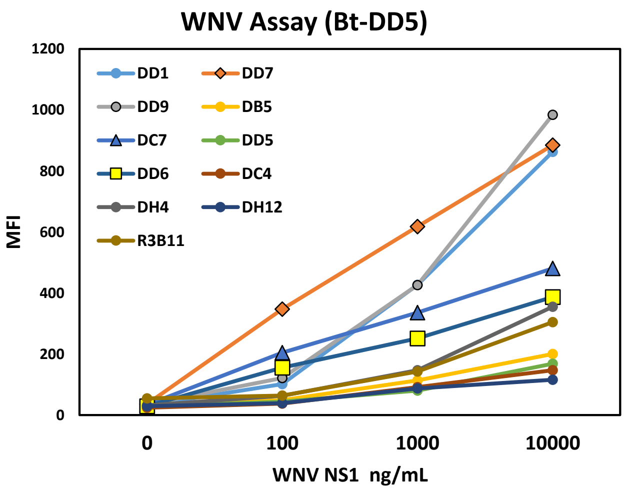

Supplemental figure S7f. MagPlex Sandwich Assays for TBEV NS1

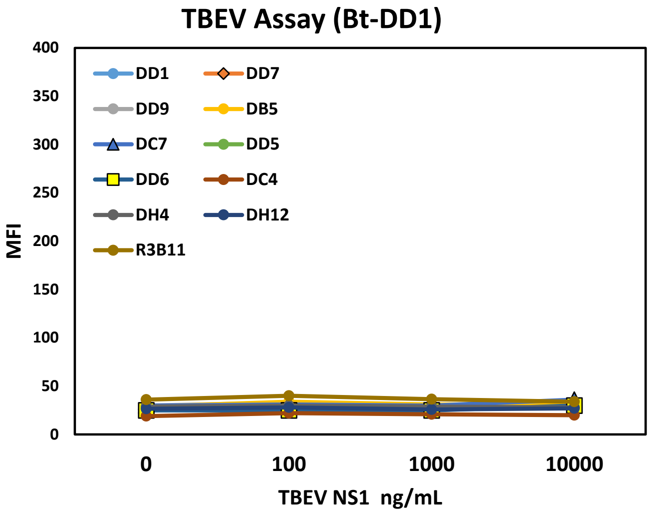

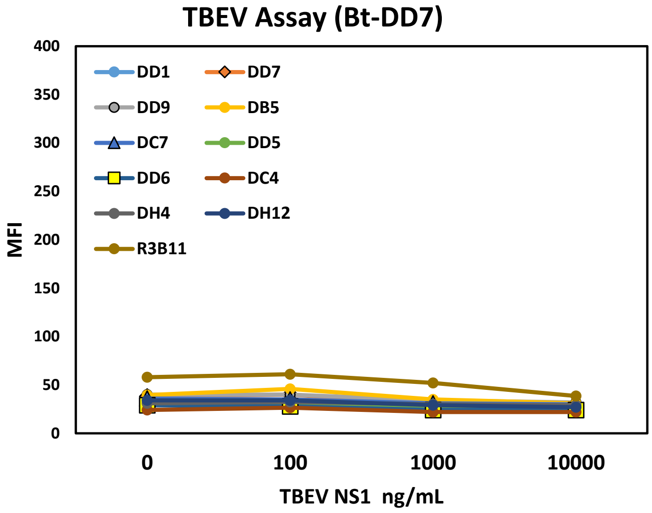

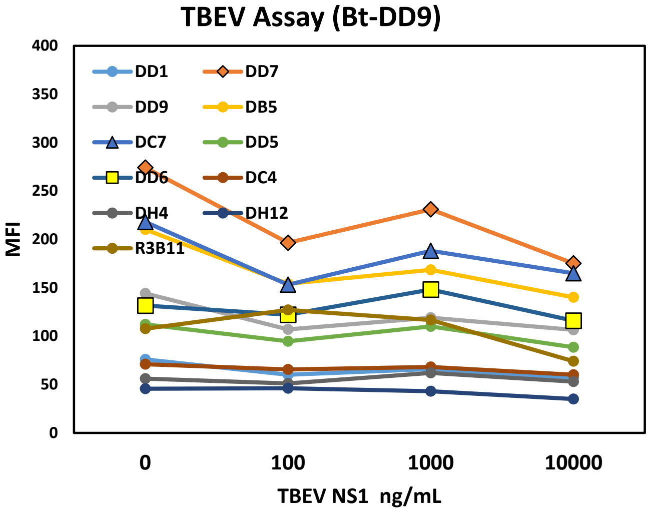

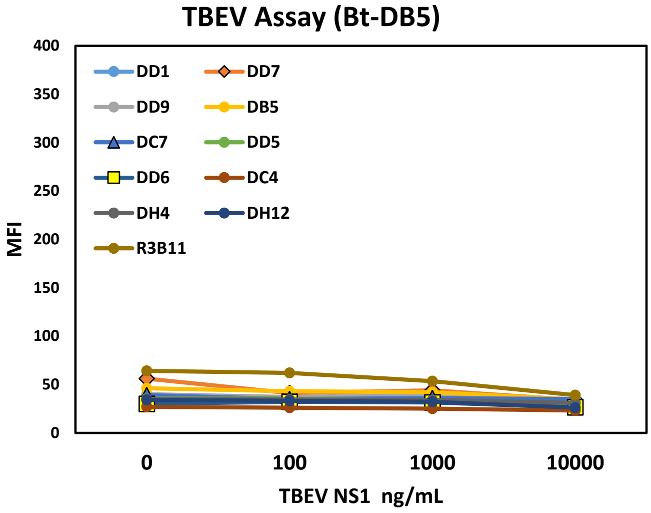

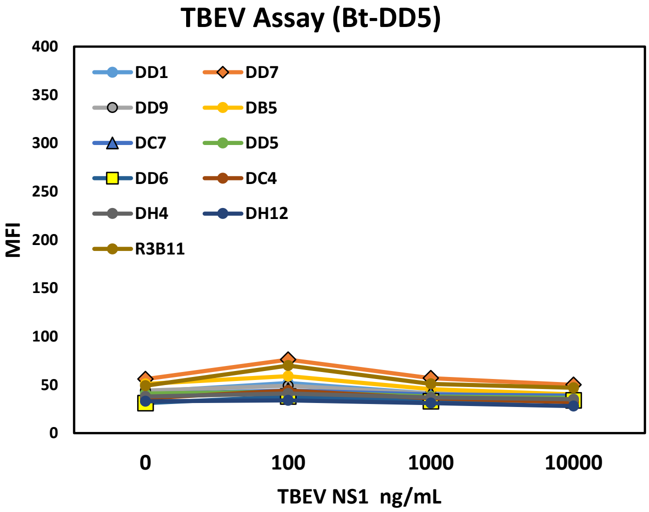

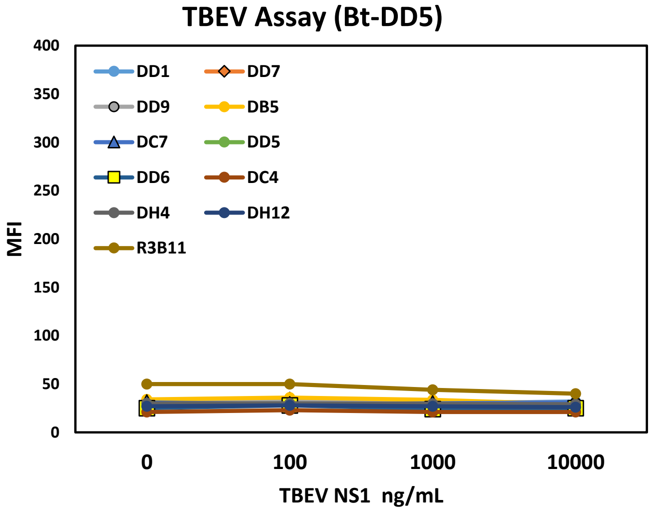

Supplemental figure S8. Sequence comparison with other NS1 variants. In all cases the strain corresponds to the one that Native Antigen Company used as the basis for their recombinantly produced NS1 product

```
          1        10        20        30        40        50        60        70        80        90        100       110       120       130
          |--------+---------+---------+---------+---------+---------+---------+---------+---------+---------+---------+---------+---------|
   DENV-1 DSGCVINWKGRELKCGSGIFVTNEVHTWTEQYKFQADSPKRLSAAIGKAWEEGVCGIRSATRLENIMWKQISNELNHILLENDMKFTVVVGDVSGILAQGKKMIRPQPMEHKYSWKSWGKAKIIGADVQN
   DENV-3 DMGCVINWKGKELKCGSGIFVTNEVHTWTEQYKFQADSPKRLATAIAGAWENGVCGIRSTTRMENLLWKQIANELNYILWENNIKLTVVVGDTLGVLEQGKRTLTPQPMELKYSWKTWGKAKIVTAETQN
   DENV-2 DSGCVVSWKNKELKCGSGIFITDNVHTWTEQYKFQPESPSKLASAIQKAHEEGICGIRSVTRLENLMWKQITPELNHILSENEVKLTIMTGDIKGIMQAGKRSLRPQPTELKYSWKTWGKAKMLSTESHN
   DENV-4 DMGCVASWSGKELKCGSGIFVVDNVHTWTEQYKFQPESPARLASAILNAHKDGVCGIRSTTRLENVMWKQITNELNYVLWEGGHDLTVVAGDVKGVLTKGKRALTPPVSDLKYSWKTWGKAKIFTPEARN
      WNV DTGCAIDISRQELRCGSGVFIHNDVEAWMDRYKYYPETPQGLAKIIQKAHKEGVCGLRSVSRLEHQMWEAVKDELNTLLKENGVDLSVVVEKQEGMYKSAPKRLTATTEKLEIGWKAWGKSILFAPELAN
Consensus D.GCvi.wkgkELkCGSG!F!t##VhtWt#qYK%qp#sP.rLa.aI.kAhe#G!CGiRS.tR$En.$Wkq!.nELN.iL.En..klt!vvgd..G.l..gkr.ltpqp.elkysWKtWGKaki...#..N

          131      140       150       160       170       180       190       200       210       220       230       240       250       260
          |--------+---------+---------+---------+---------+---------+---------+---------+---------+---------+---------+---------+---------|
   DENV-1 TTFIIDGPNTPECPDNQRAWNIWEVEDYGFGIFTTNIWLKLRDSYTQVCDHRLMSAAIKDSKAVHADMGYWIESEKNETWKLARASFIEVKTCIWPKSHTLWSNGVLESEMIIPKIYGGPISQHNYRPGY
   DENV-3 SSFIIDGPNTPECPSASRAWNVWEVEDYGFGVFTTNIWLKLREVYTQLCDHRLMSAAVKDERAVHADMGYWIESQKNGSWKLEKASLIEVKTCTWPKSHTLWTNGVLESDMIIPKSLAGPISQHNYRPGY
   DENV-2 QTFLIDGPETAECPNTNRAWNSLEVEDYGFGVFTTNIWLKLKEKQDVFCDSKLMSAAIKDNRAVHADMGYWIESALNDTWKIEKASFIEVKNCHWPKSHTLWSNGVLESEMIIPKNLAGPVSQHNYRPGY
   DENV-4 STFLIDGPDTSECPNERRAWNSLEVEDYGFGMFTTNIWMKFREGSSEVCDHRLMSAAIKDQKAVHADMGYWIESSKNQTWQIEKASLIEVKTCLWPKTHTLWSNGVLESQMLIPKSYAGPFSQHNYRQGY
      WNV NTFVVDGPETKECPTQNRAWNSLEVEDFGFGLTSTRMFLKVRESNTTECDSKIIGTAVKNNLAIHSDLSYWIESRLNDTWKLERAVLGEVKSCTWPETHTLWGDGILESDLIIPVTLAGPRSNHNRRPGY
Consensus .tF.!DGP#T.ECP...RAWNslEVED%GFG.ftTniw$Klr#..t..CDhrlmsaA!K#..A!HaD$gYWIES.kN.tWklekAsliEVKtC.WPksHTLWs#G!LES#$iIPk.laGP.S#HNyRpGY

          261      270       280       290       300       310       320       330       340       35052
          |--------+---------+---------+---------+---------+---------+---------+---------+---------+-|
   DENV-1 FTQTAGPWHLGKLELDFDLCEGTTVVVDEHCGNRGPSLRTTTVTGKTIHEWCCRSCTLPPLRFKGEDGCWYGMEIRPVKEKEENLVKSMVSA
   DENV-3 HTQTAGPWHLGKLELDFNYCEGTTVVITESCGTRGPSLRTTTVSGKLIHEWCCRSCTLPPLRYMGEDGCWYGMEIRPISEKEENMVKSLVSA
   DENV-2 HTQITGPWHLGKLEMDFDFCDGTTVVVTEDCGNRGPSLRTTTASGKLITEWCCRSCTLPPLRYRGEDGCWYGMEIRPLKEKEENLVNSLVTA
   DENV-4 ATQTVGPWHLGKLEIDFGECPGTTVTIQEDCDHRGPSLRTTTASGKLVTQWCCRSCTMPPLRFLGEDGCWYGMEIRPLSEKEENMVKSQVTA
      WNV KTQNQGPWDEGRVEIDFDYCPGTTVTLSESCGHRGPATRTTTESGKLITDWCCRSCTLPPLRYQTDSGCWYGMEIRPQRHDEKTLVQSQVNA
Consensus .TQt.GPWhlGklE.DFd.C.GTTVv..E.Cg.RGPslRTTT.sGKl!t#WCCRSCT$PPLR%.g#dGCWYGMEIRP..ekEen$VkS.V.A
```

Comparison with NS1 from WNV strain NY-99

```
          1        10        20        30        40        50        60        70        80        90        100       110       120       130
          |--------+---------+---------+---------+---------+---------+---------+---------+---------+---------+---------+---------+---------|
   DENV-1 DSGCVINWKGRELKCGSGIFVTNEVHTWTEQYKFQADSPKRLSAAIGKAWEEGVCGIRSATRLENIMWKQISNELNHILLENDMKFTVVVGDVSGILAQGKKMIRPQPMEHKYSWKSWGKAKIIGADVQN
   DENV-3 DMGCVINWKGKELKCGSGIFVTNEVHTWTEQYKFQADSPKRLATAIAGAWENGVCGIRSTTRMENLLWKQIANELNYILWENNIKLTVVVGDTLGVLEQGKRTLTPQPMELKYSWKTWGKAKIVTAETQN
   DENV-2 DSGCVVSWKNKELKCGSGIFITDNVHTWTEQYKFQPESPSKLASAIQKAHEEGICGIRSVTRLENLMWKQITPELNHILSENEVKLTIMTGDIKGIMQAGKRSLRPQPTELKYSWKTWGKAKMLSTESHN
   DENV-4 DMGCVASWSGKELKCGSGIFVVDNVHTWTEQYKFQPESPARLASAILNAHKDGVCGIRSTTRLENVMWKQITNELNYVLWEGGHDLTVVAGDVKGVLTKGKRALTPPVSDLKYSWKTWGKAKIFTPEARN
      YFV DQGCAINFGKRELKCGDGIFIFRDSDDWLNKYSYYPEDPVKLASIVKASFEEGKCGLNSVDSLEHEMWRSRADEINAIFEENEVDISVVVQDPKNVYQRGTHPFSRIRDGLQYGWKTWGKNLVFSPGRKN
Consensus D.GCvinw..kELKCGsGIF!..#vhtWt#qYk%qp#sP.kLasa!..a.e#G.CGirSvtr$En.$Wkqi..ElN.!l.Enevdlt!vvgD.kg!.q.Gkr.l.p....lkYsWKtWGKak.fspe..N

          131      140       150       160       170       180       190       200       210       220       230       240       250       260
          |--------+---------+---------+---------+---------+---------+---------+---------+---------+---------+---------+---------+---------|
   DENV-1 TTFIIDGPNTPECPDNQRAWNIWEVEDYGFGIFTTNIWLKLRDSYTQVCDHRLMSAAIKDSKAVHADMGYWIES-EKNETWKLARASFIEVKTCIWPKSHTLWSNGVLESEMIIPKIYGGPISQHNYRPG
   DENV-3 SSFIIDGPNTPECPSASRAWNVWEVEDYGFGVFTTNIWLKLREVYTQLCDHRLMSAAVKDERAVHADMGYWIES-QKNGSWKLEKASLIEVKTCTWPKSHTLWTNGVLESDMIIPKSLAGPISQHNYRPG
   DENV-2 QTFLIDGPETAECPNTNRAWNSLEVEDYGFGVFTTNIWLKLKEKQDVFCDSKLMSAAIKDNRAVHADMGYWIES-ALNDTWKIEKASFIEVKNCHWPKSHTLWSNGVLESEMIIPKNLAGPVSQHNYRPG
   DENV-4 STFLIDGPDTSECPNERRAWNSLEVEDYGFGMFTTNIWMKFREGSSEVCDHRLMSAAIKDQKAVHADMGYWIES-SKNQTWQIEKASLIEVKTCLWPKTHTLWSNGVLESQMLIPKSYAGPFSQHNYRQG
      YFV GSFIIDGKSRKECPFSNRVWNSFQIEEFGTGVFTTRVYMDAVFEYTIDCDGSILGAAVNGKKSAHGSPTFWMGSHEVNGTWMIHTLEALDYKECEWPLTHTIGTS-VEESEMFMPRSIGGPVSSHNHIPG
Consensus .tFiIDGp.t.ECP..nRaWNs.#!E#%GfGvFTTn!w$k..e.yt..CD..l$sAA!kd.kavHadmg%WieS...N.tW.iekas.i#vK.C.WPktHTlwsngVlES#M.iPks.aGPvSqHNyrpG

          261      270       280       290       300       310       320       330       340       350353
          |--------+---------+---------+---------+---------+---------+---------+---------+---------+--|
   DENV-1 YFTQTAGPWHLGKLELDFDLCEGTTVVVDEHCGNRGPSLRTTTVTGKTIHEWCCRSCTLPPLRFKGEDGCWYGMEIRPVKEKEENLVKSMVSA
   DENV-3 YHTQTAGPWHLGKLELDFNYCEGTTVVITESCGTRGPSLRTTTVSGKLIHEWCCRSCTLPPLRYMGEDGCWYGMEIRPISEKEENMVKSLVSA
   DENV-2 YHTQITGPWHLGKLEMDFDFCDGTTVVVTEDCGNRGPSLRTTTASGKLITEWCCRSCTLPPLRYRGEDGCWYGMEIRPLKEKEENLVNSLVTA
   DENV-4 YATQTVGPWHLGKLEIDFGECPGTTVTIQEDCDHRGPSLRTTTASGKLVTQWCCRSCTMPPLRFLGEDGCWYGMEIRPLSEKEENMVKSQVTA
      YFV YKVQTNGPWMQVPLEVKREACPGTSVIIDGNCDGRGKSTRSTTDSGKVIPEWCCRSCTMPPVSFHGSDGCWYPMEIRPRKTHESHLVRSWVTA
Consensus Y.tQt.GPWhlgkLE.df..CpGTtV.!.e.Cd.RGpSlRtTT.sGKl!.#WCCRSCT$PPlr%.GeDGCWYgMEIRP.kekEen$V.S.VtA
```

Comparison with NS1 from YFV strain 17D

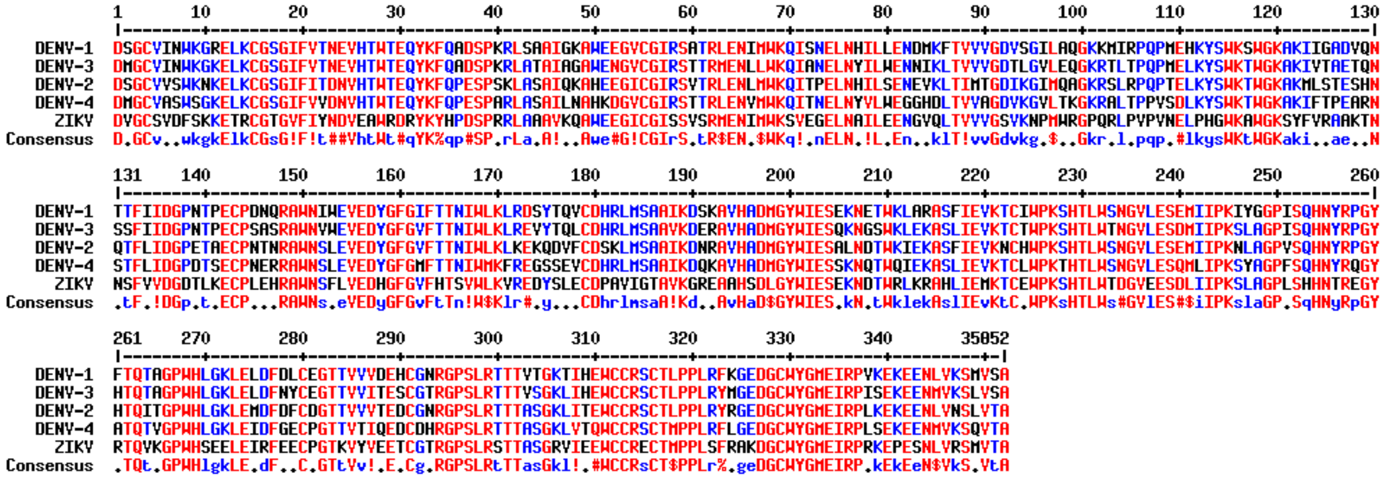

Comparison with NS1 from ZIKV strain Uganda MR 766

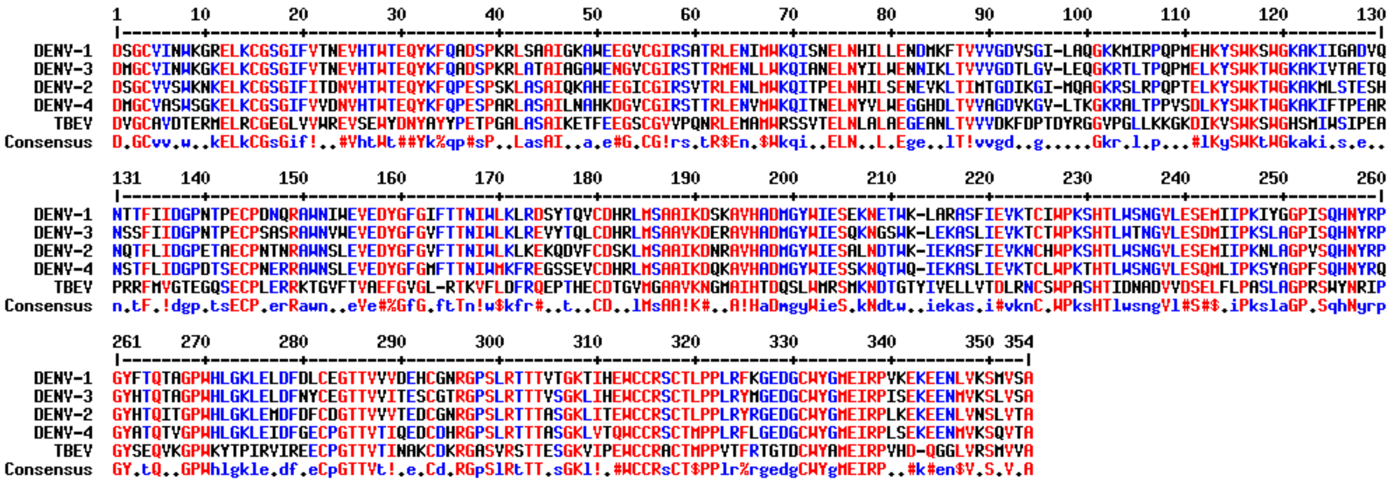

Comparison with NS1 from TBEV strain Neudoerfl

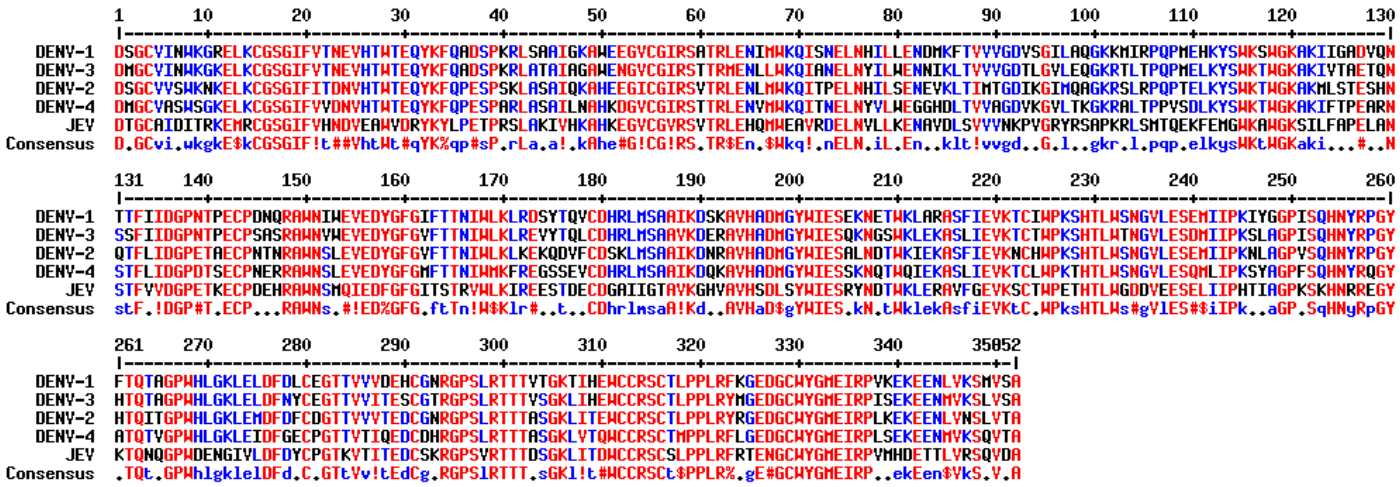

Comparison with NS1 from JEV strain SA-14

Supplemental figure S9. Blinded unknown samples of DENV NS1 spiked into serum samples. All results in the order tested. The red line indicates a ratio of 2, set as the cut-off for positive samples. Plots are shown for the duplicate samples. Key to sample number shown below the two plots.

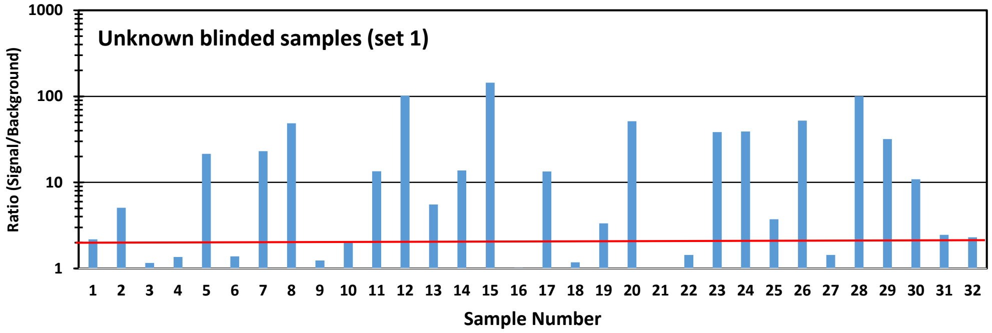

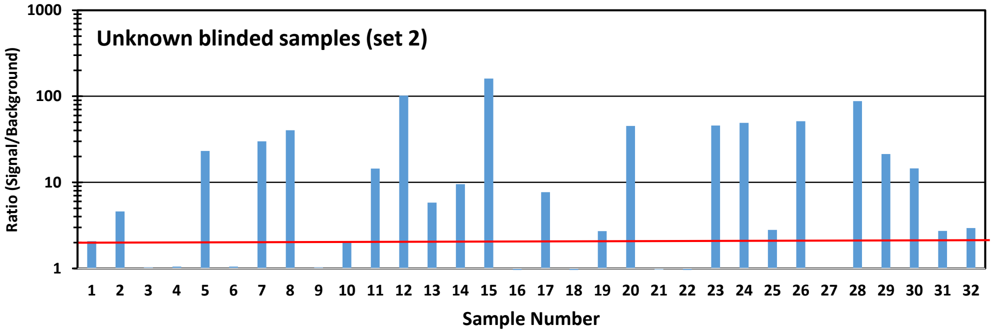

Sample #	Sample	Final Conc ng/ml	MAGPIX Results		Serum Conc
1	V1	6.25	2.18	2.075	12.5
2	V2	4	5.08	4.6	8
3	blank	0	1.16	1.025	0
4	blank	0	1.36	1.05	0
5	V3	30	21.54	23.125	60
6	blank	0	1.38	1.05	0
7	V3	30	23.06	30.0125	60
8	V4	110	48.74	40.2125	220
9	blank	0	1.24	1.025	0
10	V1	6.25	2.01	2.05	12.5
11	V1	25	13.48	14.4625	50
12	V3	120	102.22	102.125	240
13	V2	4	5.54	5.825	8
14	V4	36.6	13.74	9.525	73.2
15	V2	100	144.54	160.525	200
16	blank	0	1.02	0.925	0
17	V4	36.6	13.39	7.675	73.2
18	blank	0	1.18	0.925	0
19	V4	12.2	3.34	2.7125	24.4
20	V1	100	51.34	45.3	200
21	blank	0	1	0.975	0
22	blank	0	1.44	0.95	0
23	V2	20	38.33	45.6375	40
24	V2	20	39	49.225	40
25	V4	12.2	3.73	2.8	24.4
26	V1	100	52.14	51.3	200
27	blank	0	1.44	1.0125	0
28	V3	120	100.4	87.9875	240
29	V4	110	31.88	21.325	220
30	V1	25	10.9	14.55	50
31	V3	7.5	2.46	2.725	15
32	V3	7.5	2.3	2.9375	15

Supplemental table S1. Detection of DENV-2 NS1 at NMRC

Concentration DENV-2 NS1	Ctrl	Ctrl	Av Ctrl	Signal	Signal	Av signal	ratio	determination
89 ng/mL	32.5	34	33.25	4255	4004	4129.5	124.2	Positive
44.5 ng/mL	30	31	30.5	1845.5	1316.5	1581	51.8	Positive
22.25 ng/mL	29	28	28.5	1232	1249.5	1240.75	43.5	Positive
11.125 ng/mL	28	29	28.5	655	524.5	589.75	20.7	Positive
5.5625 ng/mL	28	27	27.5	283	270.5	276.75	10.1	Positive
2.78125 ng/mL	28	28	28	155	141	148	5.3	Positive
1.390625 ng/mL	26	27	26.5	95	82	88.5	3.3	Positive
NHS	26	28	27	26	26	26	1.0	Negative

NHS (normal human serum)
